# The effect of an exopolysaccharide probiotic molecule from *Bacillus subtilis* on breast cancer cells

**DOI:** 10.3389/fonc.2023.1292635

**Published:** 2023-11-23

**Authors:** Mai R. Nguyen, Emily Ma, Debra Wyatt, Katherine L. Knight, Clodia Osipo

**Affiliations:** ^1^ M.D./Ph.D. Program, Stritch School of Medicine, Loyola University Chicago, Maywood, IL, United States; ^2^ Department of Microbiology and Immunology, Stritch School of Medicine, Loyola University Chicago, Maywood, IL, United States; ^3^ Integrated Cell Biology Program, Stritch School of Medicine, Loyola University Chicago, Maywood, IL, United States; ^4^ Department of Cancer Biology, Stritch School of Medicine, Loyola University Chicago, Maywood, IL, United States

**Keywords:** probiotics, breast cancer, exopolysaccharide, commensal bacteria, IKK beta, STAT1

## Abstract

**Introduction:**

Many well-known risk factors for breast cancer are associated with dysbiosis (an aberrant microbiome). However, how bacterial products modulate cancer are poorly understood. In this study, we investigated the effect of an exopolysaccharide (EPS) produced by the commensal bacterium *Bacillus subtilis* on breast cancer phenotypes. Although *B. subtilis* is commonly included in probiotic preparations and its EPS protects against inflammatory diseases, it was virtually unknown whether *B. subtilis*-derived EPS affects cancer.

**Methods:**

This work investigated effects of EPS on phenotypes of breast cancer cells as a cancer model. The phenotypes included proliferation, mammosphere formation, cell migration, and tumor growth in two immune compromised mouse models. RNA sequencing was performed on RNA from four breast cancer cells treated with PBS or EPS. IKKβ or STAT1 signaling was assessed using pharmacologic or RNAi-mediated knock down approaches.

**Results:**

Short-term treatment with EPS inhibited proliferation of certain breast cancer cells (T47D, MDA-MB-468, HCC1428, MDA-MB-453) while having little effect on others (MCF-7, MDA-MB-231, BT549, ZR-75-30). EPS induced G1/G0 cell cycle arrest of T47D cells while increasing apoptosis of MDA-MB-468 cells. EPS also enhanced aggressive phenotypes in T47D cells including cell migration and cancer stem cell survival. Long-term treatment with EPS (months) led to resistance *in vitro* and promoted tumor growth in immunocompromised mice. RNA-sequence analysis showed that EPS increased expression of pro-inflammatory pathways including STAT1 and NF-κB. IKKβ and/or STAT1 signaling was necessary for EPS to modulate phenotypes of EPS sensitive breast cancer cells.

**Discussion:**

These results demonstrate a multifaceted role for an EPS molecule secreted by the probiotic bacterium *B. subtilis* on breast cancer cell phenotypes. These results warrant future studies in immune competent mice and different cancer models to fully understand potential benefits and/or side effects of long-term use of probiotics.

## Introduction

Breast cancer is the most common malignancy worldwide and is the second-leading cause of cancer-related death in the U.S (1, 2). Recent development suggests that microbial dysbiosis, an abnormal stage or maladaptation of the microbiome due to disturbances, may play a pathologic role in breast cancer (3). Numerous epidemiological studies in humans and mice both associated antibiotic use with increased breast cancer risk (4–11) while consumption of probiotics or prebiotics were associated with decreased breast cancer risk (12–17). In addition, well-known risk factors for breast cancer including age, high level of circulating estrogen, alcohol consumption, obesity, low physical activity, early menarche, high breast density, and periodontal disease have all been associated with changes in the microbiome (3, 18–23). Changes in microbial communities were observed in breast tissues, breast tumors, milk ducts, distal gut and the urinary tract (3, 19, 22, 24–28). The breast microbiome was altered in the presence of a benign or invasive breast tumor, presence of distant metastases, or treatment with chemotherapy (29). Specific microbial signatures further correlate with breast cancer subtypes as well as clinical outcome (30). Together, these data suggest that dysbiosis induced by various causes may contribute to breast cancer development and/or progression. Thus, it is not surprising that the microbiome has now been recognized as a part of the tumor microenvironment, believed to play important roles in immune suppression and/or supporting tumor growth (31).

*Bacillus subtilis* is a ubiquitous Gram-positive bacterium commonly included in commercial probiotic preparations. *B. subtilis* is also used to ferment a variety of non-dairy, traditional foods in many parts of Asia (32, 33). Although *B. subtilis* has not been studied in the context of breast cancer, it is known to secrete a variety of bioactive molecules, including antimicrobial peptides, polyketides, and bacteriocins (34). On the contrary, *B. subtilis* is the primary producer of the serine protease subtilisin, which depletes tumor suppressor proteins Deleted in Colorectal Cancer (DCC) and Neogenin in breast cancer cells, leading to enhanced migration and cancer development (35, 36).

*B. subtilis* can also form robust biofilms, which are an assembly of tightly associated bacteria encapsulated in a self-produced extracellular matrix (37). Exopolysaccharide (EPS), whether secreted into the extracellular matrix or remain bound to the cell surface, provides structural support to the extracellular matrix and is an important component in biofilm formation (38). The Knight laboratory has purified and studied exclusively EPS from *B. subtilis*. On western blots, EPS appeared as a single band at approximately 300 kDa, suggesting that EPS may be one large structure with structural analysis currently underway (39). EPS was found to have profound immunomodulatory properties via modulation of TLR4 signaling on myeloid cells (39–41). Systemic administration of EPS was found to be protective against a number of T-cell mediated inflammatory disease, including *C. rodentium* induced acute colitis, systemic *S. aureus* infection, house dust mite (HDM)-induced allergic eosinophilia, and acute Graft-versus-Host Disease (39–45).

Although a number of exopolysaccharides produced by various bacteria were tested for their anti-tumor activities *in vitro*, the majority of EPS studied were from probiotic lactic acid-producing bacteria (46–49). This study was the first to investigate effects of EPS treatment on breast cancer cells *in vitro* and *in vivo* across multiple cell lines and cancer-associated phenotypes. Our results demonstrate the complexity of EPS effects on breast cancer phenotypes from inhibiting bulk cell proliferation in the short-term to enhancing aggressive tumor phenotypes, leading to a pro-tumorigenic effect on cell-derived xenografts. Thus, bacterial molecules may influence growth properties of some types of breast cancer cells in a multifaceted manner, necessitating further studies to optimize the microbiome to benefit breast cancer prevention and treatment.

## Materials and methods

### Cell lines and culture conditions

MCF-7, T47D, MDA-MB-231, MDA-MB-453, MDA-MB-468, ZR-75-30, HCC1428, and BT549 cells were purchased from American Type Culture Collection (ATCC, Manassas, VA). Cell lines were grown in antibiotic-free Roswell Park Memorial Institute Medium (RPMI-1640, Thermo Fisher Scientific, Waltham, MA). RPMI-1640 was supplemented with 10% Fetal Bovine Serum (FBS, Gemini Bio Products, Sacramento, CA), 2mM L-glutamine (Thermo Fisher Scientific, Waltham, MA), 100µM non-essential amino acids (Invitrogen, Carlsbad, CA), and 1mM sodium pyruvate (Thermo Fisher Scientific, Waltham, MA). T47D cells were maintained in above RPMI media supplemented with penicillin (50 U/mL, Hyclone, Cat#SV30010) and streptomycin (50 μg/mL, Hyclone, Cat#SV30010) when culturing cells for long-term EPS treatments or injection in mice. All cell lines were authenticated by short tandem repeat allelic profiling (ATCC, Manassas, VA) and maintained at below 20 passages. All cells were regularly tested for mycoplasma contamination using the MycoSensor QPCR assay kit (Agilent Technologies, Santa Clara, CA). Cells were maintained in a 37°C incubation chamber at 95% O_2_ and 5% CO_2_.

### Preparation of exopolysaccharide derived from *B. subtilis*

EPS was isolated from the *B. subtilis* DK7019 strain, provided by Dr. Daniel B. Kearns of Indiana University. This strain of *B. subtilis* was genetically modified (sinR::cat tasA::cat ΔpsgB Physpank-eps) to overproduce and secrete EPS under isopropyl β-D-1- thiogalactopyranoside (IPTG)-inducible conditions while lacking gamma-polyglutamic acid (γPGA). *B. subtilis* bacteria were cultured in 1.5% Luria Bertani broth (LB, Miller formulation) to stationary phase (OD=0.6 – 0.7), then grown for 4 hours on 1.5% Luria Bertani agar plates (LB, Miller formulation) with 0.1M IPTG. Bacterial supernatant was collected in a digest solution (0.45% NaCl, 50 μg/mL DNase and 30 μg/mL RNase) and centrifuged at 9000 x g at 20°C for 20 min, twice. Supernatant was incubated in 37°C water bath for 15mins, following by digestion with 40μg/mL proteinase K at 56°C overnight. EPS was precipitated with 3-4 volume of cold ethanol at -20°C for at least 4 hours. The precipitate was pelleted by centrifugation at 13,700 x g at 4°C for 30 min, resuspended in an appropriate volume of water, and boiled at 95°C for 10 min. EPS was then purified by gel filtration on Sephacryl S-500 column (GE Healthcare). Carbohydrate-positive fractions were identified using a modified phenol sulfuric acid assay (50, 51). EPS-containing fractions were pooled and centrifuged through a Vivaspin column (Millipore, Germany) to isolate molecules larger than 30,000 kDa. Finally, EPS was dialyzed using a 10K MWCO Slide-A-Lyzer (Thermo Fisher Scientific, Waltham, MA) for 3 days, and filter sterilized using a 0.22µm PES syringe filter (Millipore, Germany). All EPS preparations were quantified for total carbohydrate concentration using a modified phenol sulfuric acid assay, assessed for the lack of protein and nucleic acid content by spectrometry, and tested for the ability to inhibit T47D proliferation prior to use.

### Drugs, antibodies and reagents

Cerdulatinib and TPCA-1 were purchased from Selleck Chemicals (Houston, TX) and suspended in 100% DMSO to a stock concentration of 1mM and stored at -80°C. Stock solutions were diluted in medium to a working concentration of 1µM. Recombinant human IFNγ protein was obtained from CellGenix (Cat# 1425-050). Matrigel Basement Membrane Matrix was purchased from Corning (Tewksbury, MA, Cat# 354234) for mice experiments. Antibodies used for flow cytometry included: PE anti-human TLR4 antibody (Biolegend, Cat# 312805), PE mouse IgG2a Kappa isotype control (Biolegend, Cat# 400211), biotin anti-mouse IgG2a antibody (Biolegend, Cat# 407103), PE Streptavidin (Biolegend, Cat# 405203). Live/Dead Fixable Aqua Stain Kit was used purchased from Invitrogen (Cat# L34957). Western antibodies STAT1 (#9172), Phosphorylated STAT1 (Tyr701, #7649), STAT3 (#9132), Phosphorylated STAT3 (Tyr705, #9131), P38 (#9212), Phosphorylated P38 (Thr180/Tyr182, #4511), P65 (#4764), Phosphorylated P65 (Ser536, # 3033) Phosphorylated IkBα (Ser32, #2859), Phosphorylated IKKα/ß (Ser176/180, #2697), and RelB (#4922) were purchased from Cell Signaling Technologies (Danavers, MA). Loading control β-Actin (A5441) was purchased from Sigma Aldrich (St. Louis, MO). Horseradish peroxidase (HRP)-conjugated secondary antibodies, including anti-rabbit (#7074) and anti-mouse (#7076) were purchased from Cell Signaling Technologies.

### RNA interference and transfection

A pool of four siRNAs was purchased from Dharmacon GE Life Sciences (Lafayette, CO) for each of the following genes: IKK-beta (ON-TARGETplus SMART pool Cat# L-003503-00-0005) and P65 (ON-TARGETplus SMART pool Cat# L-003533-00-0005). Non-targeting scrambled control siRNA (SCBi) was purchased from Qiagen (Germantown, MD). The siRNAs were reconstituted in siRNA Diluent Buffer (10mM Tris-HCl, pH 8.0, 20mM NaCl, 1mM EDTA) at 10μM working solution and stored at -20°C. The transfection reagent Lipofectamine RNAiMAX (Cat# 13778150) was purchased from Thermo Fisher Scientific (Waltham, MA) and used at a ratio of 1:1 with 50nM of appropriate siRNA according to the manufacturer’s protocol. 1.2 million T47D cells were plated in a 10-cm^2^ tissue culture overnight. The iMAX solution was prepared by adding 60μL of RNAiMAX to 940μL of Opti-MEM (per transfection) in a 2.0 mL eppendorf tube. In parallel, 60μL of siRNA was added to 940μL of Opti-MEM per transfection in separate tubes. Solutions were incubated for 5 minutes at room temperature. After incubation, 1000μL of iMAX solution was then added to each siRNA condition and allowed to incubate for 20 minutes at room temperature. The adherent cells were then washed with PBS 2X and 9mL of RPMI was added to each plate followed by 2000μL of the siRNA + iMAX solution in a drop-wise fashion. Plates were gently swirled to mix the solution and incubated at 37°C for 48 hours before splitting into experimental groups.

### Proliferation assays

Cells at a density of 50,000 were seeded in triplicate in a 6-well tissue culture plate and allowed to adhere overnight. Cells were treated with either 5µg/mL of EPS or equivalent volume of sterile PBS, and media was changed every other day. Separate wells were plated to count the number of live cells following treatment on day 2, 4 and 6. Briefly, cells in each well were trypsinized, individualized and 10µl of this cell mixture was added to 10uL of trypan blue. Live cells were counted using the Invitrogen Countess Automated Cell Counter (Hampton, NH).

### XTT survival assay

Cells at a density of 2,500 were plated into a flat-bottom 96-well tissue culture plate to adhere overnight. Cells were treated with either PBS or increasing concentrations of EPS (0 – 10,000 ng/mL), with n=6 wells per treatment. Media was changed every other day. On day 6, media was aspirated and 150uL of working XTT solution containing 0.5 mg/mL XTT (Goldbio, Cat# X-200-100) and 3.75 µg/mL Phenazine methosulfate (Sigma, Cat # P9625-1G) in phenol-red free RPMI. Plate was covered in aluminum foil and incubated at 37°C for 2h. Absorbances at 450nm (A450) and 690nm (A690) were measured using a plate reader. To calculate corrected absorbance, we subtracted (A450 - A690) of each sample with that of a blank well containing XTT solution only. Percent proliferation was calculated as [(Corrected absorbance of EPS sample/Corrected absorbance of PBS sample)*100]. Data were graphed as log(EPS concentration) versus Percent Proliferation. The log(inhibitor) vs response – Variable slope (four parameters) model on GraphPad Prism (San Diego, CA) was used to determine the IC50 (inhibitory concentration at 50%).

### Cell cycle analysis

Cells at a density of 100,000 were plated in triplicate in a 12-well tissue culture plate to adhere overnight. Cells were pretreated with stated concentrations of inhibitors or DMSO for 30min if applicable, following by treatment with either 5µg/mL of EPS or equivalent volume of sterile PBS for 24h. Cells, media, PBS wash, and trypsin solution were collected into a flow-activated cell sorting (FACS) tube and centrifuged at 500g for 5mins. The cell pellet was washed in 1mL cold PBS, centrifuged, and resuspended in 400uL of ice-cold PBS. To fix cells, 800uL of ice-cold 100% ethanol was added drop-wise under slow vortexing. Cells were stored at -20°C for at least 2 hours. On the day of analysis, cells were allowed to equilibrate to room temperature, resuspended and centrifuged at 500g at 4°C for 5min. Cells were washed once in 1mL cold PBS, and resuspended in 150µL of staining solution containing 50g/mL of propidium iodide (Sigma-Aldrich) and 10μg/mL of RNAse A in PBS. Tubes were covered with aluminum foiled and incubated for 1h at 37°C. Cell cycle analysis was conducted using LSRFortessa or FACSCantoII flow cytometers (BD Biosciences) according to the manufacturer’s instructions (Cell Signaling Technology. Data was analyzed using the Cell Cycle model on FlowJo V10 (BD Biosciences).

### Cell death analysis

Cells at a density of 100,000 were plated in triplicate in a 6-well tissue culture plate to adhere overnight. Cells were pretreated with either 5µg/mL of EPS or equivalent volume of sterile PBS for 3 days with no media change. When cells reached 80-90% confluency on the day of analysis, cells along with media, PBS wash, and trypsin solution were collected into a flow-activated cell sorting (FACS) tube and centrifuged at 1200 RPM at room temperature for 5mins. Cells were washed with cold PBS twice, and resuspended in 1mL of 1X binding buffer (10mM Hepes/NaOH, pH7.4, 140 mM NaCl, 2.5 mM CaCl2, 556454, BD biosciences, San Jose, CA). Live cells were counted using trypan blue exclusion and the Countess Cell Counter. Cells at a density of 100,000 were transferred to a new FACS tube, centrifuged and resuspended in 100µL of 1X binding buffer (BD Biosciences) containing 5µL of FITC-Annexin V (Cat# 556420, BD Biosciences, San Jose, CA) and 5µL of 7-AAD (BD Pharmingen, Cat#51-68-98E). Cells were incubated in the dark at room temperature for 15min, followed by addition of 400µL of 1X binding buffer (BD Biosciences). Cells were analyzed within 1 hour on the LSRFortessa or FACSCantoII flow cytometers (BD Biosciences) according to the manufacturer’s instructions (BD Biosciences). Data was analyzed with gating strategies to exclude debris on FlowJo V10 (BD Biosciences).

### Wound-healing migration scratch assay

Cells at a density of 200,000 were plated in triplicate in a 12-well tissue culture plate to adhere overnight. Cells were pretreated with stated concentrations of inhibitors or DMSO for 30min if applicable, following by treatment with either 5µg/mL of EPS or equivalent volume of sterile PBS for 2 days until confluent. Then cells were starved in media containing 3% FBS and drug treatments overnight. Media was aspirated and 3mL of PBS added to the well. Then a 10µL pipette tip was used to scratch the confluent monolayer of cells, creating a cross shape in the well. The scratches were immediately imaged at 2 locations of the cross at 10X objective under the microscope (0 h). Media was changed to contain 3% FBS with continued treatment of either EPS or PBS. At 24h and 48h post-scratch, media was changed and scratches were imaged at the same location relative to the cross shape. Migration rate was quantified as open gap area using ImageJ according to Venter and Niesler protocol (52). Percent wound closure was calculated as [100 - (Gap area at 24h or 48h/Gap area at 0h)*100].

### Xenograft tumor growth

All animal study protocols were approved by Loyola University’s Institutional Animal Care and Use Committee. T47D cells were expanded in 150cm^2^ tissue culture treated flasks and treated with 5μg/mL EPS or equal volume of PBS for 5 days. Then 40 million EPS or PBS-treated T47D cells were transferred to a Nunc Cell Factory System (Thermo Scientific, Cat# 140004TS) with continued treatment for another 3 days. On collection day, cells were trypsinized and resuspended in Matrigel^®^ Matrix Basement Membrane Phenol-Red Free (Cat# 356237, Corning, Bedford MA) to a concentration of 4 million live cells per 100μL of Matrigel. For EPS-treated cells, EPS was also added to the Matrigel : Cell suspension to an estimated concentration of 300μg/mL. Then 100μL of Matrigel : Cell suspension was injected bilaterally into the fourth mammary fat pads of 9-10 weeks old, female, ovariectomized Foxn1 nu/nu athymic nude mice (Envigo, IN). Mice were also implanted with a 0.3cm silastic capsule containing 17β-estradiol for a constant release of 83-100pg/mL as previously described (53). The estrogen capsule was replaced after 8 weeks. Each mouse monitored by tagging the ear with a number. Four mice per group were implanted with EPS or PBS-pretreated cells followed by intraperitoneal injection with respective 50μg EPS or 100µl PBS 3 times/week. Tumor area (length x width) was measured weekly using Vernier calipers. Mice were euthanized on day 94 and tumors were imaged, weighed, and frozen at -80 °C. Tumor growth as tumor weight and tumor volume (V=0.5×L×W^2^) were calculated and graphed.

For the experiment with NOD.SCID mice, 100 million T47D cells per condition were grown and pretreated *in vitro* with PBS or EPS for 8 days as above. On collection day, EPS-treated cells were resuspended in Matrigel with EPS added to a concentration of 80μg/mL. Four million cells were injected bilaterally into the fourth mammary fat pads of 9-10 weeks old, female, ovariectomized NOD.SCID mice (Envigo, IN). Five mice were used for PBS group and seven mice for EPS group. Each mouse was injected (i.p.) with 25μg EPS or 100µl PBS 3 times/week and tumor area (length x width) was measured weekly using Vernier calipers. Mice were euthanized on day 87 to assess tumor burden.

### RNA sequencing and pathway analysis

T47D cells (4x10^5^), MCF-7 cells (1x10^6^), MDA-MB-231 cells (2x10^5^), or MDA-MB-468 cells (8x10^5^) were plated in 10cm^2^ dishes overnight. The following day, cells at <70% confluence were treated with either 5μg/mL EPS or equal volume of PBS and incubated at 37°C for 24 hours. Each condition was performed in 3 biological replicates. Total RNA was extracted using the RNeasy mini Kit (Qiagen, Germantown, MD) and sent to Novogene for RNA-library preparation and RNA-sequencing. Novogene performed the initial analysis. Additional analysis was conducted on differentially regulated genes using the Metascape pathway analysis software (https://metascape.org), with pathway enrichment being plotted by p-value for the number of genes in a given Gene Ontology (GO) pathway.

### Statistical analysis

Experiments were conducted in triplicate and repeated at least three independent times, with results reported as Mean ± SEM. Statistical analysis was performed and figures were generated using Prism Version 9 (GraphPad Software). A two-sided Student’s *T*-test was used to compare 2 groups, and P-values <0.05 were considered statistically significant. An ANOVA with a post- hoc Tukey’s test was used to compare multiple groups. For mice studies, tumor volumes were calculated as [(LxW^2^)/2]. Linear regression analysis was performed and the slope of tumor growth over time for each treatment group was used to compare the growth rates between treatment groups.

### Lysate preparation and western blot analysis

#### Mammosphere forming assay

##### Reverse transcription and real-time polymerase chain reaction

See **Supplementary Materials and Methods** for full descriptions.

## Results

### The effect of EPS on proliferation of breast cancer cells

**Figure 1:**
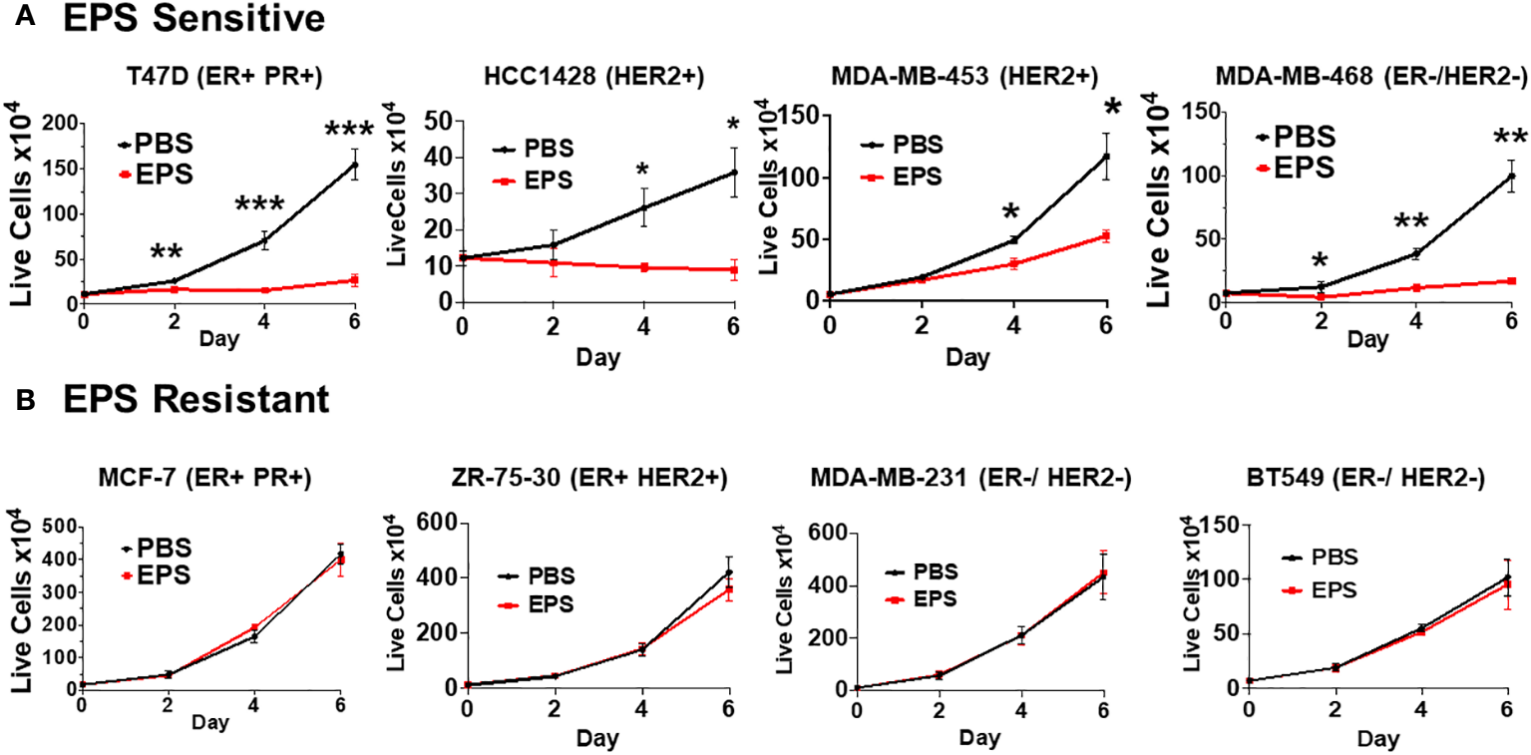
Sensitivity of different breast cancer cell lines to EPS. The proliferation rates for 8 breast cancer cell lines were measured by treating cells with PBS or 5 μg/mL EPS everyday for 6 days. **(A)** T47D, HCC1428, MDA-MB-453, and MDA-MB-468 cells were treated with PBS or EPS for 6 days. Live cells were counted and plated at day 0, and then following treatment at day 2, 4, and 6. **(B)** MCF-7, ZR-75-30, MDA-MB-231, and BT549 cells were treated and live cells counted as described in **(A)**. Data are mean values ± SEM of 3 independent experiments performed in triplicate. Statistical significance was calculated using a Student’s T-test. * P ≤ 0.05, **P ≤ 0.01 *** ≤ 0.001.

**Figure 2:**
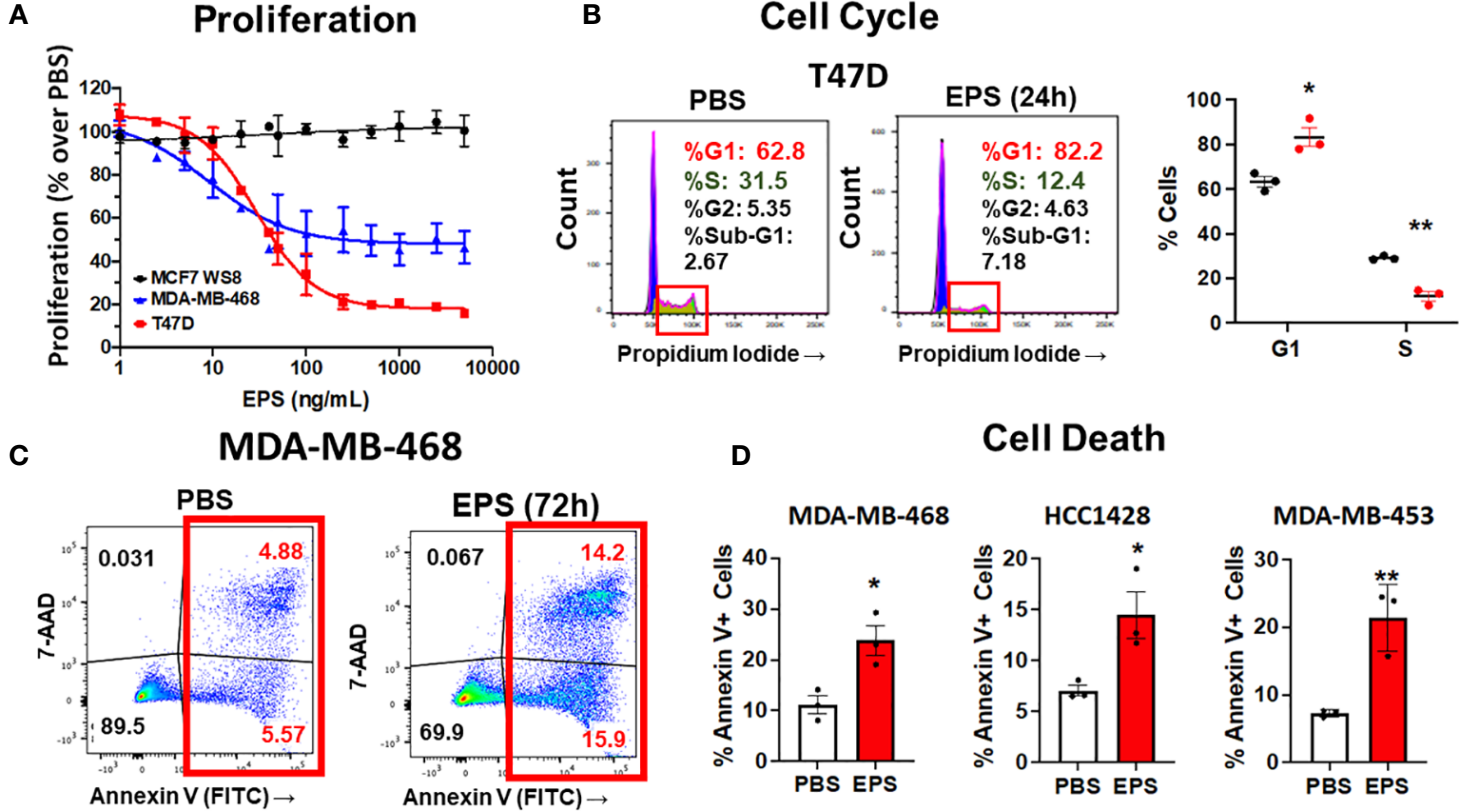
Analysis of cell cycle arrest and cell death in EPS-treated cells. **(A)** Proliferation of three breast cancer cell lines grown in medium containing PBS or increasing concentrations of EPS for 6 days. Proliferation with the PBS-treated group set at 100%. Data mean values ± SEM of 3 independent experiments. **(B)** Cells were treated with PBS or 5μg/mL EPS for 24h, stained with propidium iodide, and cell cycle analysis was performed. Representative flow cytometry plots (left) with graphical summary of 3 independent experiments performed in triplicate (right): Data are mean values ± SEM. Statistical significance was calculated using a Student’s *T*-test * P ≤ 0.05, ** ≤ 0.01. **(C)** Flow cytometric analysis of cells treated with PBS or 5μg/mL EPS for 3 days and stained with Annexin V and 7-AAD. **(D)** Percent Annexin V^+^ cells as mean ± SEM of 3 independent experiments performed in duplicate for MDA-MD-468 and HCC1428 cells and as mean ± SD of only one experiment performed in triplicate for MDA-MB-453 cells. Statistical significance was calculated using a Student’s *T*-test. * P ≤ 0.05, ** P ≤ 0.01.

Various exopolysaccharides produced by bacteria display anti-cancer activities *in vitro* (54–57). EPS produced by *B. subtilis* acts on myeloid cells to inhibit T-cell proliferation (40, 42, 44, 45). Thus, we hypothesized that EPS would inhibit the proliferation of breast cancer cells. We measured proliferation of a panel of breast cancer cells representing different subtypes (ER+PR+, HER2+, ER-HER2-, ER+HER2+) in response to PBS or 5μg/mL EPS in a time-dependent manner. Of the eight cell lines tested, four were inhibited by EPS (T47D, HCC1428, MDA-MB-453, and MDA-MB-468) (**Figure 1A**), while the rest were unresponsive (MCF-7, ZR-75-30, MDA-MB-231, and BT549) (**Figure 1B**). The sensitivity to EPS seemed to be independent of breast cancer subtypes at least based on these cell lines. To determine if sensitivity to EPS was concentration dependent, cells (T47D, MDA-MB-468, and MCF-7) were treated with increasing concentrations of EPS for 6 days, and we found that the proliferation of both T47D and MDA-MB-468 cell lines was inhibited in a concentration-dependent manner, while the MCF-7 cell line was unaffected (**Figure 2A**). Previous studies showed that TLR4 was required for biological effects of EPS on immune cells (39–41). To investigate the role of TLR4 on EPS-mediated growth inhibition of breast cancer cells, we utilized a CRISPR/Cas9 knockout approach to delete TLR4 in T47D cells. Flow cytometry showed that TLR4 is undetectable in T47D wild type, Cas9, or knockout cells (**Supplementary Figure 1A**). DNA sequencing confirmed that both alleles of the TLR4 gene had an insertion or a deletion (**Supplementary Figure 1B**), and yet EPS decreased proliferation of both wild type and TLR4 knockout cells (**Supplementary Figure 1C**). These results suggest that EPS-mediated inhibition of breast cancer proliferation is independent of TLR4.

### Cell cycle progression and cell death

Since EPS inhibited cell proliferation of some types of breast cancer cells, we hypothesized that EPS induced cell death and/or cell cycle arrest in the responsive breast cancer cells. To test these possibilities, each of the four responsive cell lines (T47D, MDA-MB-468, HCC1428, and MDA-MB-453) was treated with PBS or 5µg/mL EPS for 24 hours and assessed for cell cycle progression and cell death. EPS increased the percentage of T47D cells in the G1/G0 phase and decreased cells in the S phase (**Figure 2B**), but had little effect on cell death (**Supplementary Figure 2**). The other three cell lines (MDA-MB-468, HCC1428, MDA-MB-453), displayed minimal change in cell cycle progression in response to EPS (**Supplementary Figures 3A, 4A**, **5A**). However, EPS increased Annexin V+ MDA-MB-468 cells by 2 to 3-fold (**Figure 2C**) and similar results were observed for HCC1428, MDA-MB-453, and MDA-MB-453 cells (**Figure 2D** and **Supplementary Figures 4B, 5B**). Because of the heterogeneity of breast cancer cell lines, it was not surprising that EPS induced cell cycle arrest in some cell lines and cell death in others.

### Survival of breast cancer stem cells and cell migration in response to EPS

**Figure 3:**
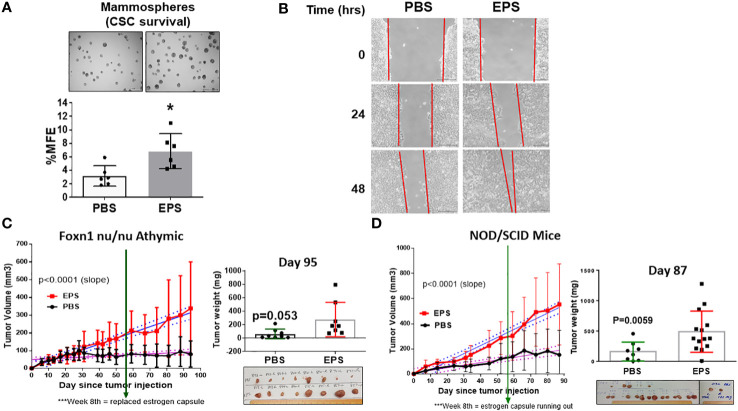
Effect of EPS on survival of cancer stem cells, migration, and tumor growth of T47D cancer cells. **(A)** Representative images (4X magnification) of mammospheres larger than 100µm and percent mammosphere forming efficiency (%MFE = # Mammospheres/25,000 Cells Plated) of T47D cells treated with PBS or 5μg/mL EPS for 4 days. Scale bar = 500µm. Data are mean values ± SD of 6 independent experiments performed as a single replicate. Statistical significance was calculated using a Student’s *T*-test. * P < 0.05. **(B)** Scratch assay of T47D cells treated with PBS or 5μg/mL EPS for 24 and 48h. Experiments were repeated at least 3 times. **(C)** T47D cells treated with 5µg/mL EPS or PBS *in vitro* for 8 days, and 4x10^6^ cells injected into mammary fat pads of four female, ovariectomized, foxn1 nu/nu, athymic nude mice implanted with a capsule releasing 17β-estradiol. EPS was i.p injected with 50µg EPS or 100µl PBS 3 times/week. Tumor volume (mm^3^) ± SD of 8 tumors per group (left). Tumor mass (mg) ± SD with a Student’s *T*-test = * P =0.053. **(D)** T47D cells treated with 5µg/mL EPS or PBS *in vitro* for 8 days, and 4x10^6^ cells injected into the mammary fat pads of female, ovariectomized, NOD/SCID mice as in **(C)** Left graph shows tumor volume (mm^3^) or tumor mass (mg) mean ± SD of 8-14 tumors per group. Student’s *T*-test was used to assess statistical significance between slopes or mass.

A thorough investigation of any new cancer agent should include assessment not only of proliferation, but also of other cancer-associated phenotypes, including survival of cancer stem cells and cell migration. We tested if EPS affected breast cancer stem cells (BCSCs), or tumor-initiating cells, a small population of cells within bulk tumors displaying stem-cell properties. These cells are capable of self-renewal, differentiation along mammary epithelial lineages, proliferation, and clonal nonadherent spherical clusters (mammosphere formation) (58, 59). Due to these stem-like characteristics, BCSCs are thought to be responsible for treatment resistance, recurrence and metastasis (60–68). We utilized the mammosphere formation assay, which assesses BCSCs based on their ability to survive and proliferate in a 3D culture, and tested if EPS altered the survival of BCSCs. Surprisingly, pretreatment of bulk T47D cells with EPS increased mammosphere forming efficiency by nearly 2 fold compared to control PBS-treated cells (**Figure 3A**).

The wound-healing scratch assay was performed on T47D cells to measure their migration capacity in response to EPS, and these cells showed increased cell migration compared to PBS-treated cells (**Figure 3B**). These results suggest that although EPS induces G0/G1 cell cycle arrest of T47D cells, it paradoxically enhanced survival of BCSCs and increased their rate of migration.

### Effect of EPS on growth of T47D tumor xenografts in athymic, nude and NOD/SCID mice

To determine the physiological role and implication of long-term EPS treatment on breast tumor growth, we first utilized an orthotopic xenograft model in which ER+ T47D human breast cancer cells were injected into the mammary fat pads of female athymic, nude mice. Mice from each group (N=4) were treated with PBS or 50µg EPS via intraperitoneal (i.p) injection thrice weekly. EPS treatment significantly increased the rate of tumor growth in nude mice, although it did not significantly increase the mass of tumors (**Figure 3C**). In numerous other studies, EPS has been shown to induce an anti-inflammatory state, and we considered the possibility that EPS indirectly promotes tumor growth by inducing a tolerogenic immune state. Although nude mice lack a functional thymus, they have a functional innate immune compartment as well as extrathymic T cell development. As EPS is known to impact myeloid cells (39–41), we tested the effect of EPS on tumor growth using a more immunocompromised mouse model, NOD/SCID that lacks innate immune function. In experiments similar to those with the athymic, nude mice, EPS treatment increased both the rate of tumor growth and tumor mass (**Figure 3D**). These data suggest that increased tumor growth of T47D cells following long-term and frequent exposure to EPS is possibly due to intrinsic effects of EPS on breast cancer cells.

### Global gene expression profiling and pathway analysis

**Figure 4:**
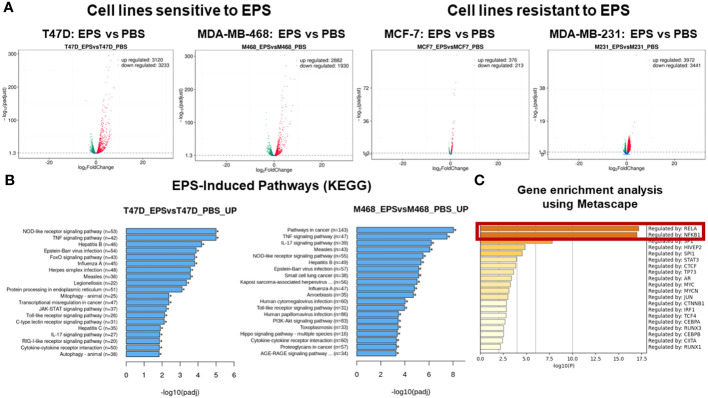
RNA-SEQ analysis of genes and pathways altered by EPS. RNA-SEQ was performed on total RNA extracted from T47D, MDA-MB-468, MCF-7, and MDA-MB-231 cells treated with 5µg/mL EPS or PBS for 24 hours. **(A)** Volcano plots were generated showing –log2 fold decrease (green) or increase (red) in expression of genes in response to EPS compared to PBS as calculated using FPKM values and –log10 padjusted values for statistical significance. **(B)** Enriched pathways for EPS compared to PBS were determined using KEGG pathway analysis. The Y-axis depicts the pathways and the X-axis shows the –log10 padjusted values. **(C)** Metascape gene enrichment analysis was performed on 290 genes-identified as being upregulated by EPS only in the sensitive but not in resistant cell lines. The data represent the -log10 p-values on the X-axis and transcriptional regulators on the Y-axis. The p-values were calculated based on 3 biological replicates.

**Figure 5:**
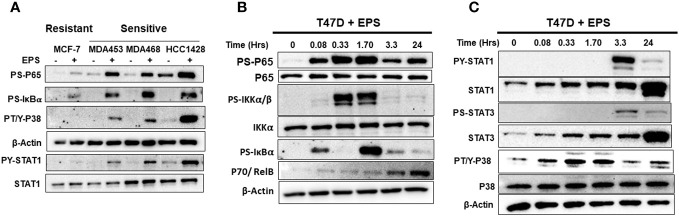
Western blot analysis of EPS activation of IKK-NF-κB, p38, and STAT1/3 pathways. **(A)** EPS-sensitive (MDA-MB-453, MDA-MB-468, and HCC1428) and resistant (MCF-7) cells were treated with PBS (–) or 5µg/mL EPS (+) for 3h. Total cell lysates were analyzed using antibodies against P-p65, P-IκBα, P-p38, β-Actin, P-STAT1, and total STAT1. **(B)** T47D cells were treated with 5µg/mL EPS for up to 24h and cell lysates were analyzed using antibodies against P-p65, total p65, P-IκBα, P-IKKα/β, total IKKα, p70, and β-Actin. **(C)** T47D cells was treated with 5µg/mL EPS for up to 24h and total cell lysates were analyzed using antibodies against indicated P-STAT1, total STAT1, P-STAT3, total STAT3, P-p38, total p38, and β-Actin. Experiments were repeated 2-3 times. Representative images for each detected protein are shown.

We employed an unbiased approach to discover mechanisms by which EPS modulates phenotypes of breast cancer cells. We aimed to identify genes and pathways altered by EPS in sensitive cells, but not in resistant cells. RNA-sequence analysis (RNA-SEQ) was performed on two sensitive cell lines (T47D and MDA-MB-468) and two resistant cell lines (MCF-7 and MDA-MB-231) treated 20 hr with PBS or EPS. Volcano plots for 3 biological replicates showed that EPS induced expression of more genes in EPS-sensitive cells than EPS-resistant cells (**Figure 4A**). KEGG pathway analysis of RNA-SEQ data showed that the top pathways altered in EPS-treated T47D cells were DNA replication and G1 transition, in agreement with the G1 cell cycle arrest induced by EPS. In addition, pathways related to bacterial/viral infection and immune responses were among the top pathways altered by EPS, including interferon and TNF signaling (**Figure 4B**). We hypothesized that EPS activates critical pathways leading to observed phenotypes and identified 290 genes that were upregulated by EPS in the sensitive but not resistant cell lines. Gene enrichment analysis was performed on this set of genes using the Metascape pathway analysis software. The canonical NF-κB was the top transcriptional regulator of these genes (**Figure 4C**). Together, these data suggest that EPS activates an inflammatory response in sensitive breast cancer cells, possibly through activation of TNF, interferon/JAK-STAT, and/or NF-κB signaling. We tested this possibility by treating cells with EPS and performing western blot analysis to identify phosphorylated proteins. Using three EPS sensitive cells (MDA-MB-453, MDA-MB-468, and HCC1428) and one resistant cell line (MCF-7), we found that EPS induced considerable phosphorylation of p65, IκB, p38, and STAT1 in sensitive cells, but little to none in the resistant cell line (**Figure 5A**). Additionally, EPS increased phosphorylation of p65, IKKα/β, IκB, and RelB within 5 min to 1.7hrs (**Figure 5B**), p38 within 5min, and STAT1 and STAT3 within 3.3hrs (**Figure 5C**) in T47D cells. The activation of canonical NF-κB, as indicated by phosphorylation of p65, occurred within 5 min of EPS treatment. Activation of STAT1 and STAT3 required at least 3hrs. These data suggest that EPS may first activate the IKK-NF-κB pathway, followed by subsequent activation of STAT1.

### Requirement of IKK signaling

**Figure 6:**
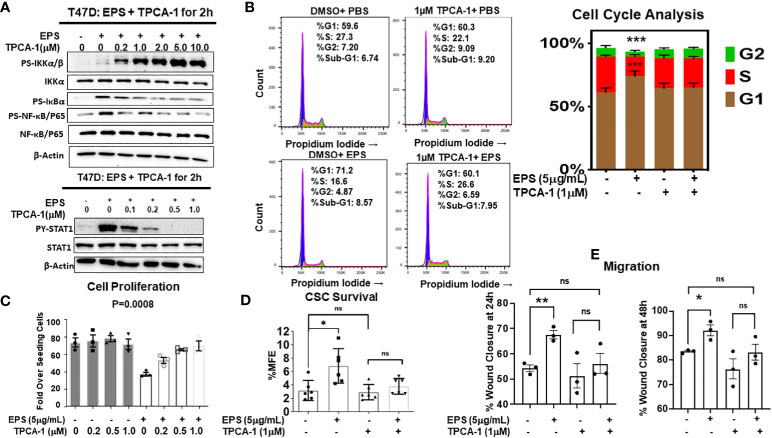
Rescue of EPS-Induced signaling and cancer associated phenotypes in T47D cells by the IKKβ inhibitor, TPCA-1. **(A)** T47D cells were pretreated with increasing amounts of TPCA-1 for 30min, then 5µg/mL EPS or PBS was added for 2h. Total cell lysates were analyzed by western blots using antibodies against: Top panel (P-IKKα/β, total IKKα, P-IκBα, P-p65, total p65, and β-actin) and Bottom panel (P-STAT1, total STAT1, P-p38, total p38 and β-actin). Experiments were repeated 3 independent times. Representative images are shown. **(B)** T47D cells were treated with PBS or 5µg/mL EPS in the presence of 1µM TPCA1 for 24h. Cells were analyzed by flow cytometry after fixing and staining with propidium iodide. Experiments were performed three independent times. Representative images are shown and the bar graph depicts data as mean ± SEM of 3 independent experiments performed in triplicate, with Student’s *T*-test comparing %S of PBS vs EPS, *** P ≤ 0.001. **(C)** Growth of T47D cells after treatment with PBS or 5µg/mL EPS, and increasing doses of TPCA-1 every 2 days for 6 days. Proliferation was calculated as in **Figure 1**. Data are represented as mean ± SEM of 3 independent experiments each performed in triplicate. A one-way ANOVA was performed, with P=0.0008 for EPS compared to the PBS control. **(D)** Percent mammosphere forming efficiency (%MFE = # Mammospheres/25,000 cells plated) of T47D cells pretreated with 1µM TPCA-1 for 30mins before PBS or 5µg/mL EPS treatment for 4 days. Data are represented as mean ± SD of 6 independent experiments, with statistical significance of P < 0.05 as calculated using a Student’s *T*-test (Left). **(E)** Scratch migration assay of T47D cells pretreated with 1µM TPCA-1 for 30mins followed by PBS or 5μg/mL EPS for 24 and 48h. Data are represented as mean ± SEM of 3 independent experiments each performed in triplicate, with Student’s *T*-test * P ≤ 0.05, ** P ≤ 0.01, ns, not statistically significant.

We tested if the IKK-NF-kB pathway is required for EPS’s effect on the sensitive cell lines by using TPCA-1, a potent inhibitor of IκB kinases (IKKs). TPCA-1 has 22-fold selectivity for IKKβ over IKKα with an IC50 of 17.9 (69), and although well-known as an IKK/NF-κB inhibitor, TPCA-1 also inhibits STAT3 (70). We treated T47D cells with increasing concentrations of TPCA-1 in the presence of PBS or EPS for 2 hrs, and by western blot analysis found that TPCA-1 reduced phosphorylation of IκBα and p65 (**Figure 6A**, upper panel), but increased phosphorylation of IKKα/β, both in a concentration-dependent manner (**Figure 6A**). These data suggest that EPS may inhibit an upstream phosphatase in the NFκB pathway. Surprisingly, TPCA-1 decreased EPS-induced STAT1 phosphorylation in a concentration-dependent manner (**Figure 6A**, lower panel), while having little effect on p38 phosphorylation (not shown), indicating that the effect on STAT1 is specific (**Figure 6A**).

Since TPCA-1 prevented EPS-mediated activation of both NF-κB and STAT1, we tested if NF-κB and/or STAT1 are required for EPS inhibition of proliferation and for the G1/G0 cell cycle arrest of T47D cells. We found that TPCA-1 (1µM) almost completely rescued the G1/G0 cell cycle arrest induced by EPS in T47D cells (**Figure 6B**), as well as the inhibition of proliferation (**Figure 6C**). Additionally, EPS-mediated upregulation of BCSCs (**Figure 6D**) and increased cell migration (**Figure 6E**) were inhibited by TPCA-1. Although TPCA-1 was very efficient at rescuing these phenotypes induced by EPS, the mechanism of action is potentially multifaceted as TPCA-1 inhibits the activation of both IKK-NF-κB and STAT1 in response to EPS.

**Figure 7:**
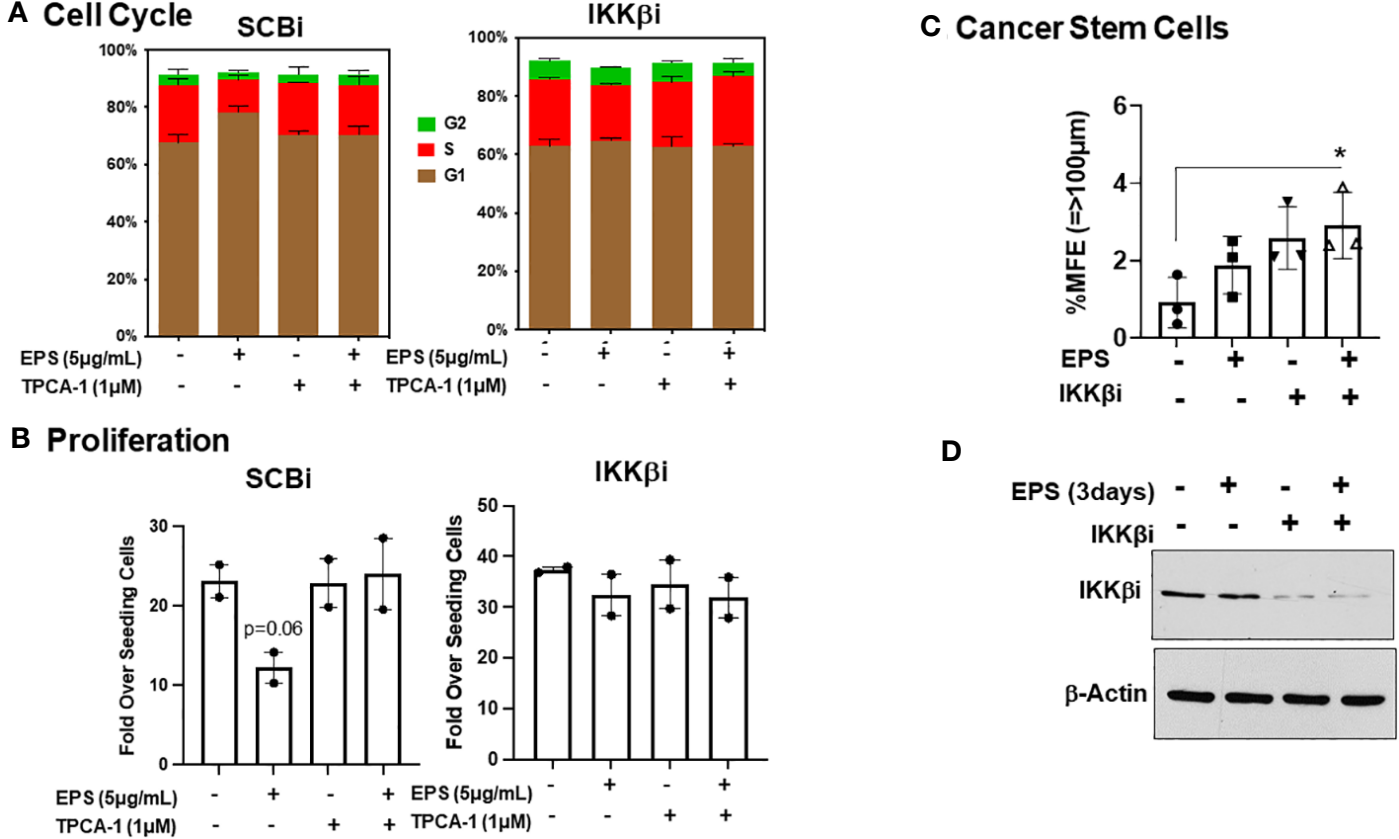
Role of IKKβ for EPS-mediated effects in T47D cells. T47D cells were transfected with IKKβ siRNA or scrambled siRNA (SCRi). **(A)** Transfected cells were plated in 12-well plates and treated with PBS or 5µg/mL EPS in the presence of DMSO or 1µM TPCA1 for 24h. Cells were fixed and stained with propidium iodide. Cell cycle analysis was performed with FlowJo. Data are represented as mean ± SEM of 2 independent experiments performed in duplicate. **(B)** Growth assay was performed on transfected cells in the presence of 5μg/mL EPS and TPCA1 for 6 day. Live cells were counted by trypan blue exclusion on a hemocytometer. Proliferation was calculated as Fold over seeding cells = (# Live Cells on Day 6)/(# Live Cells Plated on Day 0). Data are represented as mean ± SEM of 2 independent experiments performed triplicate. A T-test was performed for significance with p=0.06. **(C)** Percent mammosphere forming efficiency (%MFE = # Mammospheres/25,000 Cells Plated) of transfected T47D cells treated with PBS or EPS for 3 days. Data are represented as mean ± SD of 3 independent experiments, with statistical significance of P < 0.05 as calculated using a one-way ANOVA. **(D)** Western blot of lysates of transfected T47D cells after 3 days treatment with PBS or EPS. Blot probed with anti-IKKβ and Actin.

TPCA-1 is highly specific for IKKs, with higher selectively for IKKβ over IKKα, and we hypothesized that IKKβ maybe the direct target of TPCA-1 in EPS-treated cells. In addition, TPCA-1 potently inhibited EPS-induced STAT1 phosphorylation, suggesting that it could inhibit a kinase responsible for phosphorylating STAT1. JAK1, the upstream kinase of STAT1, is another potential target of TPCA-1 as it has been shown to inhibit JAK1 (71). To test how EPS was functioning, we performed an RNAi-mediated knockdown of IKKβ or JAK1 in T47D cells and measured cell cycle progression and proliferation in response to EPS without or with TPCA-1. IKKβ knockdown alone modestly enhanced the % of cells in S-phase and abrogated the inhibitory effects of EPS similar to TPCA-1 (**Figure 7A**). EPS-mediated inhibition of proliferation of T47D cells was rescued by IKKβ knockdown or treatment with TPCA-1 (**Figure 7B**). The effect of EPS and TPCA-1 on proliferation was due primarily to IKKβ and not to JAK1 as the knockdown of JAK1 had little effect on inhibition of proliferation by EPS nor on the rescue by TPCA-1 (**Supplementary Figure 6**). In addition, IKKβ knockdown alone increased BCSC survival and EPS had little effect when IKKβ was depleted (**Figure 7C**). Western blot analysis confirmed that IKKβ was knocked down by the siRNA (**Figure 7D**). These data indicate that the most likely target of TPCA-1 was IKKβ as it was required for EPS-mediated inhibition of proliferation and cell cycle arrest.

### The role of STAT1 signaling in EPS-mediated cell cycle arrest

Neither genetic knockout nor knockdown approaches were successful at depleting STAT1 or at preventing EPS-mediated STAT1 phosphorylation (data not shown). Hence, to address the role of STAT1 in EPS-induced changes to T47D cells, a pharmacologic approach was taken to inhibit STAT1 indirectly by targeting its upstream kinase, JAK1 using Cerdulatinib. Another target of this inhibitor is IKK, which is required by EPS to induce cell cycle arrest. This inhibitor at 1µM successfully inhibited STAT1 phosphorylation in EPS-treated T47D cells (**Supplementary Figure 7A**), but had no effect on NF-κB activation as measured by levels of phosphorylated IκB and p65 (**Supplementary Figure 7A**). Cerdulatinib (1µM) also rescued the G1/G0 cell cycle arrest induced by EPS (**Supplementary Figure 7B**). These data suggest that STAT-1 also contributes to EPS-mediated cell cycle arrest of breast cancer cells.

## Discussion

**Figure 8:**
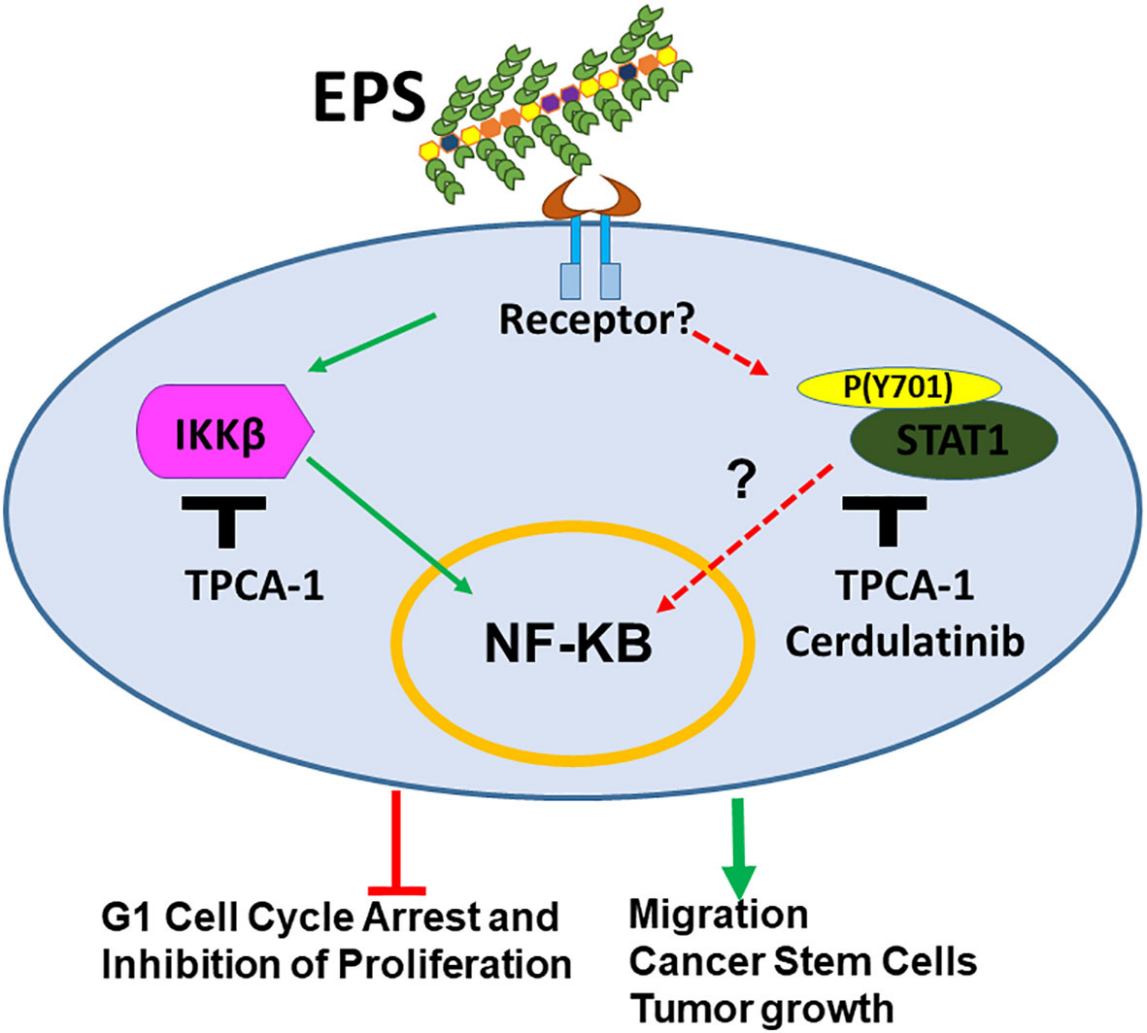
Model for mechanism by which EPS-derived from the probiotic *B. subtilis* modulates breast cancer associated phenotypes. EPS binds an unknown receptor on the cell surface activating IKKβ and STAT1 signaling. This activation leads to inhibition of cell cycle progression and proliferation of bulk cells. In addition, EPS-mediated activation of these pathways enhances cell migration, survival of cancer stem cells, and tumor growth in immunocompromised mice.

The microbiome has been recognized as being part of the tumor microenvironment. Dysbiosis induced by various factors is associated with breast cancer development (31). Microbiome studies report large-scale changes in bacterial composition, which makes it difficult to pinpoint the specific causal microbes. So far, there have been few reports regarding effects of specific commensal bacteria on breast cancer phenotypes. This study is the first to evaluate the effect of EPS produced from the commonly used probiotic strain *B. subtilis* on cancer cells, using breast cancer as a model. Although most of the work focused on T47D cells, similar results were also shown in other cell lines. We found that EPS directly modulated various phenotypes of breast cancer cells, from cell cycle arrest, inhibition of bulk cell proliferation, increased migration, increased BCSC survival, and increased tumor growth. Overall, EPS has differential activity on breast cancer cells that does not require TLR4, unlike previous studies showing that TLR4 signaling is required on myeloid cells for the anti-inflammatory effect of EPS (39–41). The receptor for EPS on breast cancer cells is yet to be identified. We performed RNA-SEQ analysis across multiple cell lines and focused on top pathways shared by sensitive and not resistant cell lines. STAT1 and IKK were activated across all four sensitive cell lines. Hence, the mechanism by which EPS exerts these effects on breast cancer cells is most likely through activation of IKKβ-NFκB signaling and possibly also STAT1 activation as shown in our current model (**Figure 8**). The NF-κB pathway was activated within minutes of EPS exposure. IKKβ inhibitors (TPCA-1 and Cerdulatinib) abrogated EPS-induced STAT1 phosphorylation and subsequent cancer associated phenotypes. IKKβ knockdown also seemed to rescue EPS-mediated growth inhibition. However, both genetic knockout or knockdown approaches directed at STAT1 were unsuccessful at completely depleting STAT1 (data not shown). Incomplete knockdown was also not useful because the small amount of STAT1 protein remaining was phosphorylated in response to EPS (data not shown). These data suggest that EPS may not utilize the canonical Interferon/JAK/STAT1 pathway to modulate breast cancer phenotypes as activation by interferon-γ did not induce cell cycle arrest and knocking down JAK1 did not interfere with EPS-mediated inhibition of proliferation. Instead, IKKβ may be associated with STAT1 phosphorylation at tyrosine 701. Since IKKβ is a serine/threonine kinase that phosphorylates IκBα (72), it is unlikely that IKKβ would be able to directly phosphorylate the tyrosine 701 on STAT1. Thus, an unidentified tyrosine kinase that is not JAK1 may be involved. IKKα, which is the sister kinase to IKKβ within the IKK complex, may also need to be investigated to see whether it plays a role in EPS signaling. We elected to knockdown IKKβ first because TPCA-1 has a 22-fold selectivity for IKKβ over IKKα (69). Interestingly, one study showed that silencing of IKKα significantly decreased STAT1 tyrosine phosphorylation in response to dsRNA in HeLa cells, suggesting that IKKα can mediate both type I interferon-dependent and interferon-independent STAT1 phosphorylation (73). However, no physical interaction between IKKα and STAT1 was detected (73). Future studies will focus on further delineating the interaction between IKKβ and STAT1 induced by EPS.

It is also important to understand which bacteria are beneficial or harmful for cancer phenotypes, and in which context. Probiotics, or the use of living microorganisms to promote health, have proven benefits (13). Several probiotics (mainly *Lactobacillus* and *Bifidobacterium* strains) have beneficial effects on prevention and treatment of breast cancer (13, 74–76). Probiotic supplements significantly reduced the incidence of chemotherapy-related cognitive impairment and alleviated gastrointestinal toxicity induced by chemotherapy or radiation in breast cancer patients (77, 78). Probiotic bacteria such as *Akkermansia muciniphila* improved response to anti-PD-1 immunotherapy (79, 80). However, other studies showed that there was little benefit from probiotic use in improving diarrhea associated with radiation or chemotherapy (54). Additional reports showed that long term probiotic use interferes with the gut commensal bacteria and may result in sepsis, fungemia and GI ischemia (55). Therefore, it will be important to understand which types of probiotics or molecule they secrete are beneficial or harmful in regards to cancer therapy.

Our results suggest a novel finding in which a well-established probiotic, commensal bacterium, *Bacillus subtilis* produces an EPS molecule that can directly alters breast cancer cell signaling and modulate breast cancer cell phenotypes. EPS has potent anti-inflammatory effects (39–45). While EPS appeared as a potent anti-proliferative agent across commonly used *in vitro* assays including viability assays (XTT), cell cycle progression, cell proliferation, and Annexin-V cell death analysis, EPS unexpectedly enhanced cell migration, BCSC survival, promoted tumor growth in immune compromised xenograft models. There are certainly more factors at play *in vivo* that could alter the tumor’s response to a drug, from drug bioavailability to other cell extrinsic phenotypes. It is also important to note that the duration of exposure to EPS is critical for phenotypes. Longer treatment in mice led to tumor growth while shorter exposure *in vitro* predominantly inhibited proliferation. These results indicate that EPS has multifaceted functions depending on the breast cancer cells and cellular environment and future studies are needed to fully elucidate the different mechanisms of action.

In the modern world where clean/urbanized environment and processed foods are common, exposure to *B. subtilis* is from unconventional sources such as fermented soybeans called Natto\Miso in Japan or Cheongukjang in Korea, or fermented cabbage called Kimchi in Korea (32, 33). *B. subtilis* has been isolated from the ileum and feces of healthy humans, and can persist in the gut for up to 20 days after its withdrawal from the diet according to animal studies (56, 57, 81). Although it is unknown if *B. subtilis* can be found in breast tissue, EPS produced by *B. subtilis* may exert local and systemic effects on the immune system, creating a healthy anti-inflammatory state as a commensal bacterium. EPS may also travel to breast tissue, interacting directly with breast cancer cells to modulate their growth and phenotypes. Additional experiments are needed to determine the physiological relevance of EPS on breast cancer and benefit to risk ratio of using this probiotic, EPS-derived from *B. subtilis.*

## Data availability statement

The data presented in this study was deposited into the NCBI database SRA (https://www.ncbi.nlm.nih.gov/sra), accession number PRJNA1036683, and in the GEO repository (https://www.ncbi.nlm.nih.gov/geo), accession number GSE248119.

## Ethics statement

The animal study was approved by Institutional Animal Care and Use Committee. The study was conducted in accordance with the local legislation and institutional requirements.

## Author contributions

MN: Data curation, Methodology, Visualization, Writing – review & editing, Conceptualization, Formal Analysis, Investigation, Writing – original draft. EM: Data curation, Investigation, Writing – review & editing. DW: Data curation, Investigation, Methodology, Validation, Writing – review & editing. KK: Writing – review & editing, Funding acquisition, Resources, Supervision, Visualization. CO: Funding acquisition, Resources, Supervision, Visualization, Writing – review & editing, Data curation, Methodology, Project administration, Validation.
