# Supplementary material for: The effect of an exopolysaccharide probiotic molecule from Bacillus subtilis on breast cancer cells
Source: Front Oncol. 2023 Nov 23;13:1292635. doi: 10.3389/fonc.2023.1292635 (PMC10702531; doi:10.3389/fonc.2023.1292635)
Supplement: Supplementary file 1 [file DataSheet_1.docx]

**Supplementary Materials and Methods:**

**Reagents**

Recombinant human TNF⍺ protein with carrier (Cat # 210-TA-020), neutralizing anti-TNF⍺ antibody (Cat # MAB610-100), and neutralizing anti-TNFRI antibody (Cat # MAB225-100) were purchased from R&D Systems. STAT1 (ON-TARGETplus SMART pool Cat# L-003543-00-0005), JAK1 (ON-TARGETplus SMART pool Cat#L-003145-00-0005),

**Lysate Preparation and Western Blot Analysis**

Following experimental treatment, 200,000 cells/well were plated in a 12-well culture treated plate overnight. Cells were treated with PBS or EPS for indicated times, then the plate was placed on ice for lysate collection. Cells had their media aspirated, washed with cold PBS twice, and finally lysed in 150ul of Triton X-100 lysis buffer containing 50mM HEPES pH 7.4, 1% Triton X-100, 150mM NaCl, 5mM EDTA, 1mM Na3VO4, 10mM NaF, 1mM PMSF, protease inhibitor cocktail (Thermo Scientific, Cat#32963). Cell lysate were scraped, collected in an Eppendorf tube, and incubated on ice for 20 mins. Next, lysates were sonicated for 10 seconds at 20% amplitude, twice using the Sonic Dismembrator (Model 100, Thermo Fisher Scientific, Waltham, MA). 2ul of each sample lysate was used to determine the protein concentration using the BCA protein assay according to manufacturer’s protocol (Thermo Fisher Scientific, Cat # 23225). The BCA plate was incubated at 37 ⁰C for 30 minutes, then each well’s absorbance at 562nm (A562) was measured on a 96-well plate fluorescent plate reader. Protein concentrations were calculated based on the linear regression of the protein standards: y=mx+b or sample protein concentration (x) = [(A562(y) – b)/m]. 20-30µg aliquots of lysate were prepared using 2X or 4X Laemmli buffer (BioRad, Hercules, CA, Cat # 1610737/1610747) and β-mercaptoethanol (Thermo Fisher Scientific, Waltham, MA, Cat# BP-176-100). Before running on western get, samples were denatured for 10 minutes at 95⁰C. 20-30µg of lysates were separated on a 10% SDS-PAGE gel buffered with 8% tris-glycine. HiMark Prestained protein standard (Thermo Fisher Scientific, Waltham, MA, Cat# LC5699) was used as molecular ladder. Proteins were run at 150V for 60 minutes, and transferred to a nitrocellulose membrane at 100V for 60 minutes. Following transfer, the membrane was blocked in 5% non-fat milk diluted in Tris Buffered Saline with Tween 20 (TBST = 5mM Tris-HCL, 5mM Tris-base, 150mM sodium chloride, 0.05% Tween-20 and 0.2% NP-40 at pH 8.0). Blocking was performed for 1 hour at room temperature under constant agitation. Then, primary antibody of interested was incubated with the membrane at 4⁰C overnight under constant agitation. All primary antibodies were used at 1:1000 dilution in 5% bovine serum albumin (BSA) in TBST, except Phospho-P38 (1:2000 in 5% BSA) and ß-actin (1:3000 in 5% milk). The next day, the membrane was washed 3 times in 1x TBST solution for 10 minutes under constant agitation at room temperature. Then, HRP-conjugated secondary antibody was diluted in 5% milk in TBST to appropriate concentration (anti-rabbit 1:1000 and anti-mouse 1:3000) and added to the membrane. The membrane was incubated with secondary antibody for 1 hour at room temperature under agitation, then washed 3 times with TBST for 10 minutes each. Finally, proteins were detected using Enhanced Chemiluminesence (Thermo Fisher Scientific) or SuperSignal West Extended Duration substrate (Thermo Fisher Scientific) at 1:1 volume. Membrane was incubated with above substrate for a few minutes. Stained bands were visualized with recommended exposure time on ProteinSimple machine (Biotechne, San Jose, CA). Membranes were stripped and re-probed multiple times. To strip, the membrane was first washed in TBST for 10 minutes, then stripped twice in mild stripping buffer (1L of buffer at pH 2.2 containing 15g glycine, 1g SDS, and 10mL Tween 20) for 5 minutes at room temperature under constant agitation. Then the membrane was washed twice in PBS for 10 minutes, and then twice in TBST for 5 minutes. After blocking in 5% milk for an hour, the membrane was ready for re-probing with primary antibody.

**Mammosphere Forming Assay**

Preparation of Mammosphere Medium: For each batch of mammosphere media, we added 196 mL of warm DMEM-F12 medium (Gibco, Gaithersburg, MD, Cat. 11039021) to a sterile bottle containing 4 grams of methocellulose. The solution was initially stirred in a water bath at 60oC for 30mins, then it was allowed to continue mixing overnight at 4oC. The next day, we added 4 mL B-27 supplement and 4μL recombinant hEGF (Sigma-Aldrich, Milwaukee, WI, Cat. E-9644) to the medium and stirred the solution for 30mins at room temperature. The solution was transfereed to 50mL centrifuge tubes and centrifuged at 8000 RPM in Beckman rotor for 30mins at 4oC. The supernatant was poured into 50mL conical tubes and stored at -20^o^C until used. The mammosphere medium was thawed in a bead bath at 37^o^C for 2-3h prior to use. 100,000 T47D cells were plated in a 6-well tissue culture plate to adhere overnight. Then cells pretreated with stated concentrations of inhibitors or DMDO for 30mins if applicable, following by treatment with either 5µg/mL of EPS or equivalent volume of sterile PBS for 4 days. Cells were harvested using trypsin and individualized. Live cells were counted using trypan blue and 25,000 cells were plated in 6-well ultra-low attachment plate with 3mL of mammosphere medium containing gentamycin. The whole plate was gently rocked several times to evenly distribute the cells across the well. The plate was left undisturbed in the incubator at 37oC and 5% CO2 for 7 days to allow mammospheres to form. On day 7, mammospheres were first imaged at 4X objective on a microscope. To extract mammospheres for counting, 6mL of PBS was added to the well and pipetted a few times to mix with the viscous mammosphere medium. Mixture containing mammospheres were transferred to a 15mL conical tube. This process was repeated with another 6mL of PBS. Next, this tube was weighted on a scale to estimate total volume. After inverting the tube multiple times to mix, 375µL was transferred to a 96-well plate and allowed to settle for 5min. Mammospheres in the 96-well plate was imaged at 4X objective along with measurement scale under a microscope. 5 pictures were taken for each well to ensure the whole well was captured. These pictures were placed into PowerPoint, and mammospheres larger than 100microns (or 50microns in some cases) were counted manually based on the scale stamped in the picture. Based on the total volume weighted, the diluted factor was calculate to estimate the total number of mammospheres present in the sample based on the mammospheres counted. Finally, percent mammosphere forming efficiency (%MFE) was calculated as [(total number of mammospheres/ 25,000 cells plated)*100].

**Reverse Transcription and Real-Time Polymerase Chain Reaction**

2x10^5^ T47D cells were plated in 6 cm2 dishes and allowed to adhere overnight. The following day, cells were treated with their respective conditions and incubated at 37°C for 24h. Plate was placed on ice, and cells were washed 2X with cold 1X PBS. Then 300μL of TRIzol® (Thermo Fisher Scientific, Waltham, MA) was added to each well to resuspend the cells. The cell mixture was collected in a 1.5mL Eppendorf tube and 50μL of 1- bromo-3-chloropropane (BCP) was added to each sample (Sigma Aldrich, St. Louis, MO) and vortex for 10 seconds. Samples were centrifuged at 15,000rpm for 15 minutes at 4°C. The clear, aqueous phase was carefully isolated and transferred to a new 1.5mL Eppendorf tube. To this tube, equal volume of 100% EtOH was added and the solution was gently vortexed. The total RNA from the sample was collected using the RiboPureTM Kit (Thermo Fisher Scientific). The sample was passed through a filter cartridge by centrifugation at 16,000rcf for 30s at room temperature. Each sample was washed with 400μL of Direct-zolTM RNA Prewash and flow-through was discarded. 5μl of DNAse I in 75μl of DNAse digestion buffer (RiboPureTM Kit, Thermo Fisher Scientific) was added directly onto the column. After 15min of incubation at room temperature, 400μL of Direct-zolTM RNA Prewash was added. The column was centrifuged and flow-through discarded. Samples were washed again with 200μL of Direct-zolTM RNA Prewash and spun for 30s. Finally, 500μL of RNA wash buffer was added and column was spun for 2min. The tubes were spun one more time at 16,000rcf for 1 minute to discard any excess buffer. The column containing RNA was moved to a new 1.5mL Eppendorf tube, and 30μL of DNase/RNase-free water was gently added to the column membrane. Samples were incubated at room temperature for 1 minutes and spun a final time at 16,000rcf for 1 minute to elute RNA. Total RNA quality and quantity was determined by measuring the UV absorbance at 260nm using the NanoDrop 1000 Spectrophotometer (Thermo Fisher Scientific). Isolated RNA was converted to cDNA using the TaqMan Reverse Transcriptase Kit (Applied Biosystems, Ford City, CA) according to manufacturer’s protocol. Briefly, 0.5μg of RNA was added to a 50μL reaction volume consisting of 1X RT buffer, 5.5mM MgCl2, 500μM dNTPs, 2.5μM random hexamers, 0.4 U/μL RNase inhibitor, and 1.25 U/μL RT enzyme (MultiscribeTM Reverse Transcriptase Enzyme, Applied Biosystems). The reverse transcriptase reaction was ran in a thermocycler as followed: 10 minutes at 25°C, 30 minutes at 48°C, 5 minutes at 95°C, 60 minutes at 25°C, and held at 4°C until use. RT-PCR was performed using the iTaqTM SYBR® Green Enzyme Supermix with ROX (BioRad, Hercules, CA) according to manufacturer’s protocol. In a 96-well optical PCR plate, 1.25μL of cDNA was added to 11.25μL of master-mix solution containing 50μM forward and reverse primers, RNase-free water, and 2x SYBER® Green. Each condition was performed in duplicate. The RT-PCR reaction was ran using a StepOnePlus thermocycler (Applied Biosystems, Foster City, CA) as followed: initial denature at 95°C for 10 minutes, PCR cycling for 10 seconds at 95°C for 40 cycles, and annealing for 45 seconds at 60°C. Melt curves were performed to ensure proper amplicon formation and the average cycle threshold (CT) was used to determine the relative gene expression for each experimental condition. The CT value was calculated as the number of cycles necessary for the fluorescent signal to overcome the background level, or threshold, of fluorescent signal. CT values were normalized to the housekeeping gene hypoxanthine-guanine phosphoribosyltransferase (HPRT), an endogenous control, to discern ΔCT. ΔCT for gene of interest= CT (experimental gene)-CT (HPRT). ΔΔCT was calculated by normalizing the ΔCT values to a control sample. Relative quantification (RQ) was calculated using the 2-ΔΔCT method to determine relative fold increases or decreases in transcript compared to the designated control sample.
