# Supplementary material for: The effect of an exopolysaccharide probiotic molecule from Bacillus subtilis on breast cancer cells
Source: Front Oncol. 2023 Nov 23;13:1292635. doi: 10.3389/fonc.2023.1292635 (PMC10702531; doi:10.3389/fonc.2023.1292635)
Supplement: Supplementary file 2 [file Presentation_1.pptx]

## Slide 1
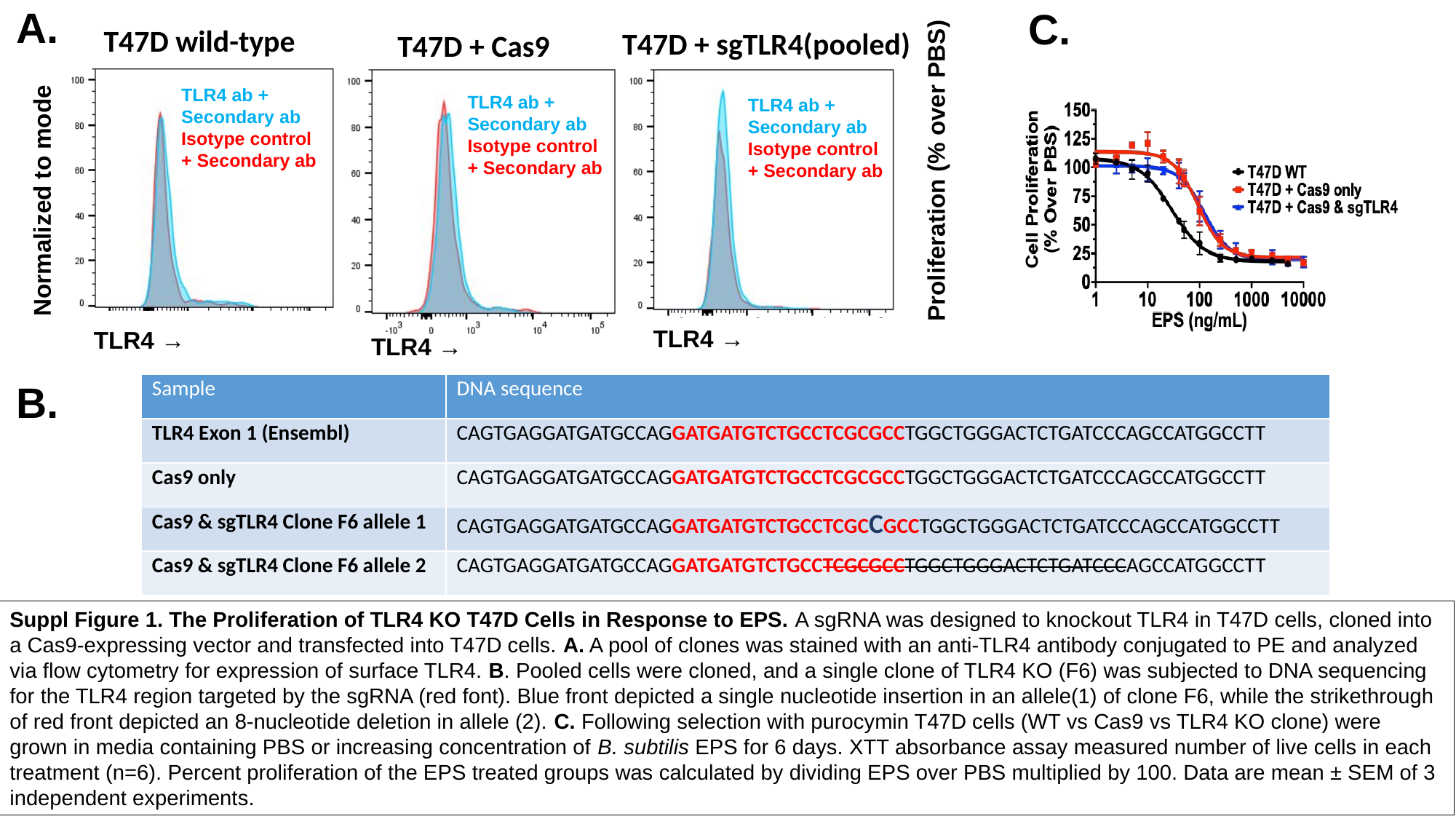

A.
C.
Proliferation (% over PBS)
T47D wild-type
T47D + sgTLR4(pooled)
T47D + Cas9
TLR4 ab + Secondary ab
Isotype control + Secondary ab
TLR4 ab + Secondary ab
Isotype control + Secondary ab
TLR4 ab + Secondary ab
Isotype control + Secondary ab
Normalized to mode
TLR4 →
TLR4 →
TLR4 →
B.
| Sample | DNA sequence |
| --- | --- |
| TLR4 Exon 1 (Ensembl) | CAGTGAGGATGATGCCAGGATGATGTCTGCCTCGCGCCTGGCTGGGACTCTGATCCCAGCCATGGCCTT |
| Cas9 only | CAGTGAGGATGATGCCAGGATGATGTCTGCCTCGCGCCTGGCTGGGACTCTGATCCCAGCCATGGCCTT |
| Cas9 & sgTLR4 Clone F6 allele 1 | CAGTGAGGATGATGCCAGGATGATGTCTGCCTCGCCGCCTGGCTGGGACTCTGATCCCAGCCATGGCCTT |
| Cas9 & sgTLR4 Clone F6 allele 2 | CAGTGAGGATGATGCCAGGATGATGTCTGCCTCGCGCCTGGCTGGGACTCTGATCCCAGCCATGGCCTT |
Suppl Figure 1. The Proliferation of TLR4 KO T47D Cells in Response to EPS. A sgRNA was designed to knockout TLR4 in T47D cells, cloned into a Cas9-expressing vector and transfected into T47D cells. A. A pool of clones was stained with an anti-TLR4 antibody conjugated to PE and analyzed via flow cytometry for expression of surface TLR4. B. Pooled cells were cloned, and a single clone of TLR4 KO (F6) was subjected to DNA sequencing for the TLR4 region targeted by the sgRNA (red font). Blue front depicted a single nucleotide insertion in an allele(1) of clone F6, while the strikethrough of red front depicted an 8-nucleotide deletion in allele (2). C. Following selection with purocymin T47D cells (WT vs Cas9 vs TLR4 KO clone) were grown in media containing PBS or increasing concentration of B. subtilis EPS for 6 days. XTT absorbance assay measured number of live cells in each treatment (n=6). Percent proliferation of the EPS treated groups was calculated by dividing EPS over PBS multiplied by 100. Data are mean ± SEM of 3 independent experiments.

## Slide 2
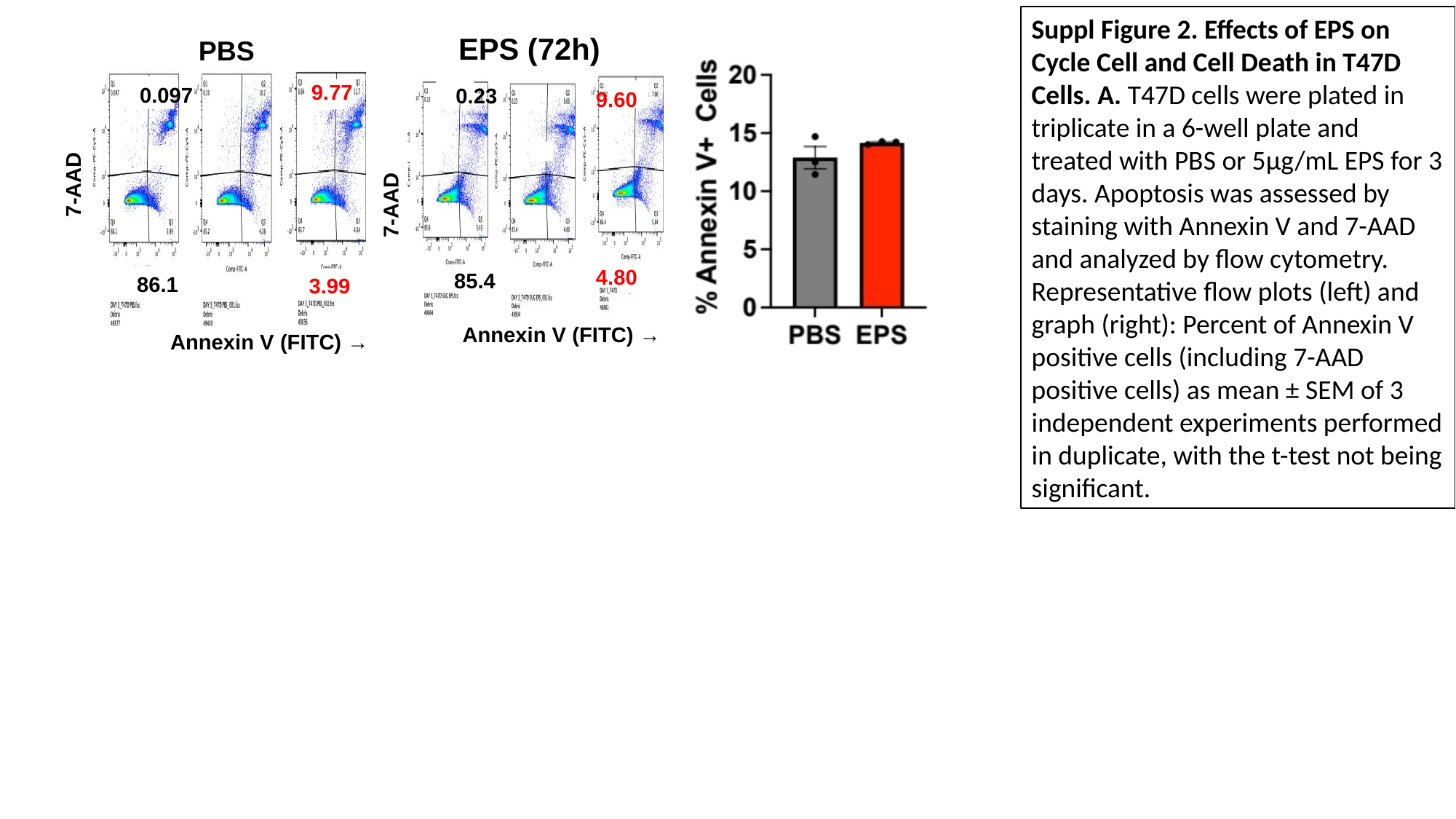

Suppl Figure 2. Effects of EPS on Cycle Cell and Cell Death in T47D Cells. A. T47D cells were plated in triplicate in a 6-well plate and treated with PBS or 5μg/mL EPS for 3 days. Apoptosis was assessed by staining with Annexin V and 7-AAD and analyzed by flow cytometry. Representative flow plots (left) and graph (right): Percent of Annexin V positive cells (including 7-AAD positive cells) as mean ± SEM of 3 independent experiments performed in duplicate, with the t-test not being significant.
EPS (72h)
PBS
9.77
0.097
0.23
9.60
7-AAD
7-AAD
4.80
85.4
86.1
3.99
Annexin V (FITC) →
Annexin V (FITC) →

## Slide 3
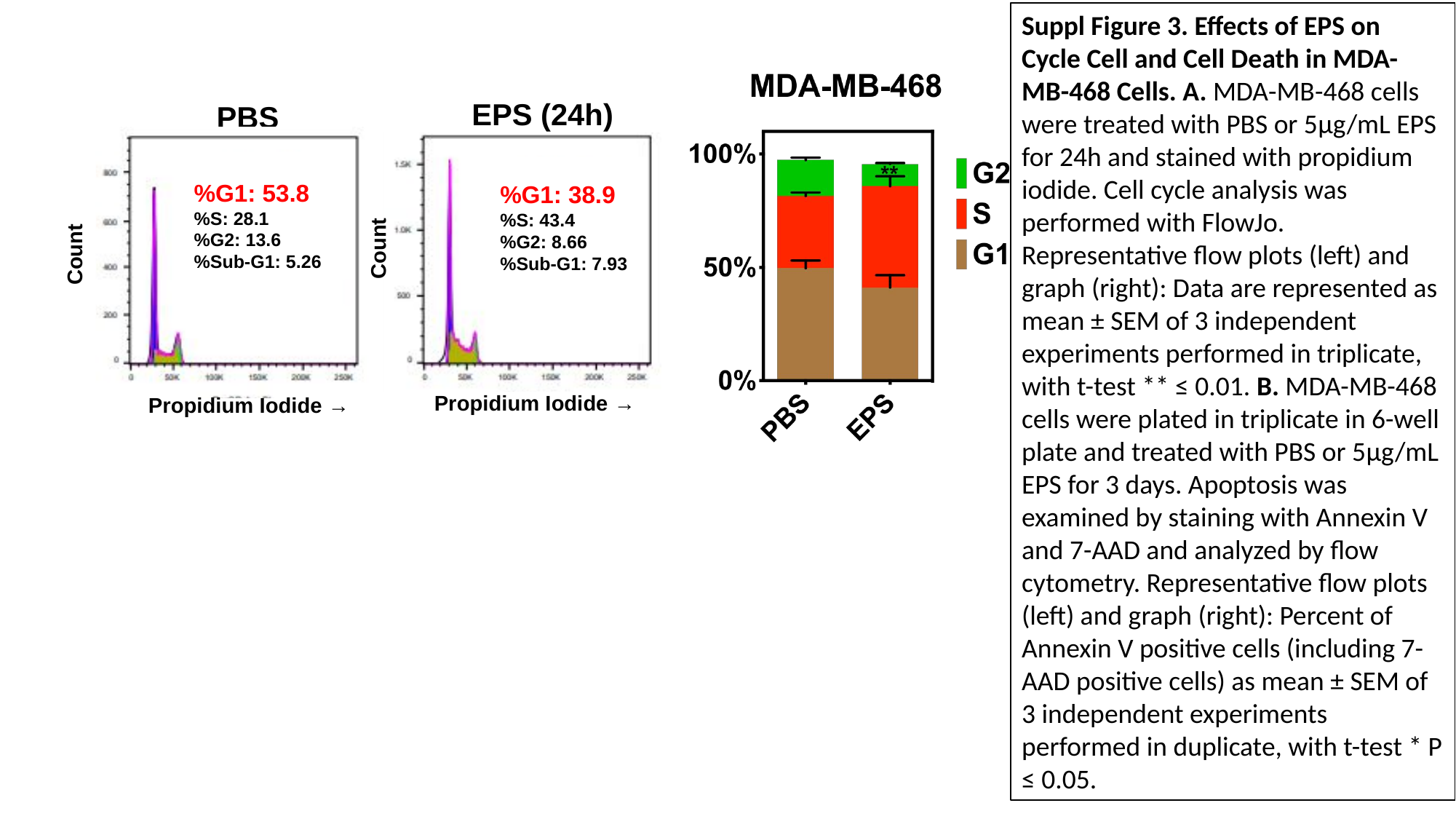

Suppl Figure 3. Effects of EPS on Cycle Cell and Cell Death in MDA-MB-468 Cells. A. MDA-MB-468 cells were treated with PBS or 5μg/mL EPS for 24h and stained with propidium iodide. Cell cycle analysis was performed with FlowJo. Representative flow plots (left) and graph (right): Data are represented as mean ± SEM of 3 independent experiments performed in triplicate, with t-test ** ≤ 0.01. B. MDA-MB-468 cells were plated in triplicate in 6-well plate and treated with PBS or 5μg/mL EPS for 3 days. Apoptosis was examined by staining with Annexin V and 7-AAD and analyzed by flow cytometry. Representative flow plots (left) and graph (right): Percent of Annexin V positive cells (including 7-AAD positive cells) as mean ± SEM of 3 independent experiments performed in duplicate, with t-test * P ≤ 0.05.
EPS (24h)
PBS
%G1: 53.8
%S: 28.1
%G2: 13.6
%Sub-G1: 5.26
%G1: 38.9
%S: 43.4
%G2: 8.66
%Sub-G1: 7.93
Count
Count
Propidium Iodide →
Propidium Iodide →

## Slide 4
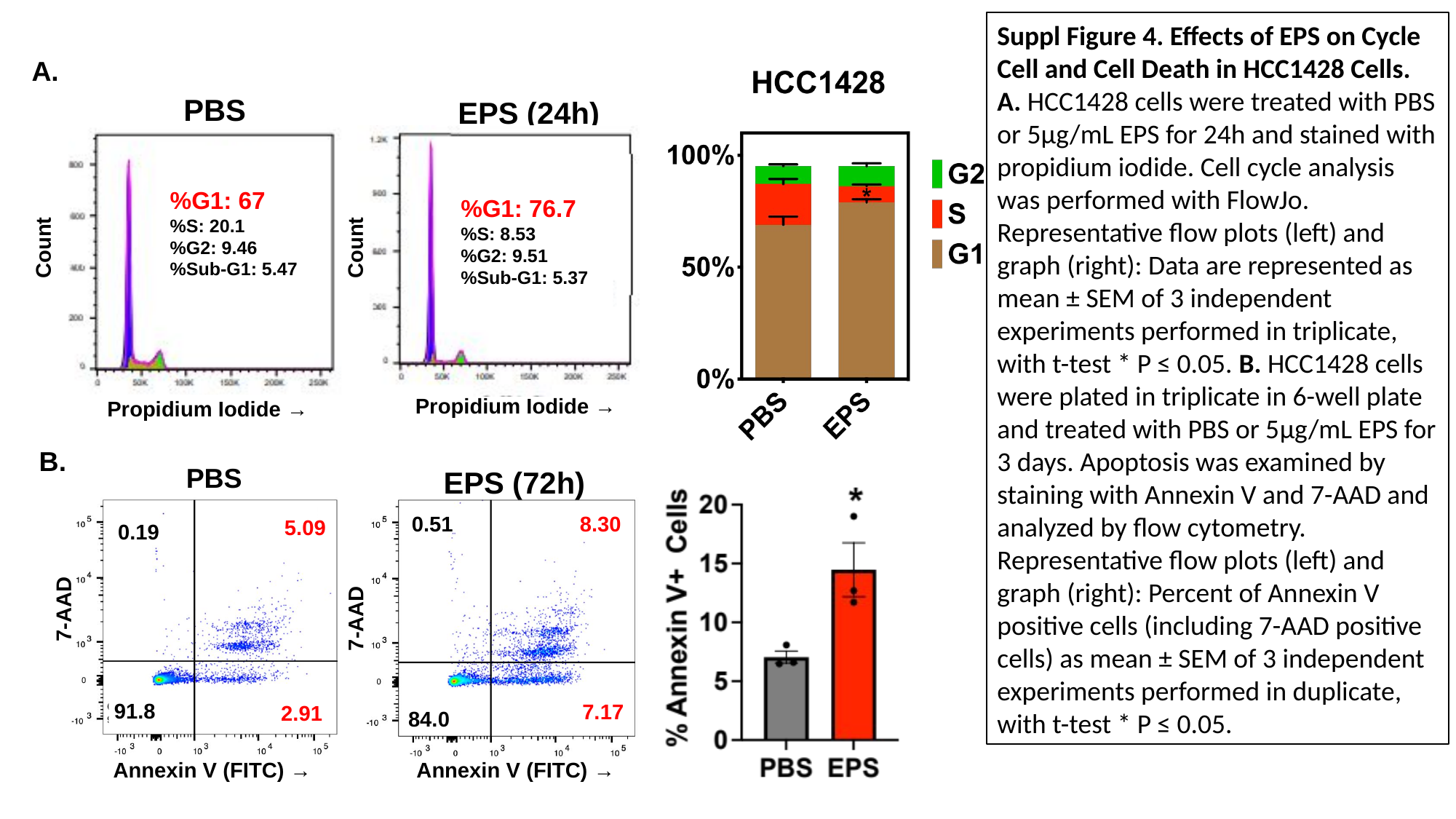

Suppl Figure 4. Effects of EPS on Cycle Cell and Cell Death in HCC1428 Cells. A. HCC1428 cells were treated with PBS or 5μg/mL EPS for 24h and stained with propidium iodide. Cell cycle analysis was performed with FlowJo. Representative flow plots (left) and graph (right): Data are represented as mean ± SEM of 3 independent experiments performed in triplicate, with t-test * P ≤ 0.05. B. HCC1428 cells were plated in triplicate in 6-well plate and treated with PBS or 5μg/mL EPS for 3 days. Apoptosis was examined by staining with Annexin V and 7-AAD and analyzed by flow cytometry. Representative flow plots (left) and graph (right): Percent of Annexin V positive cells (including 7-AAD positive cells) as mean ± SEM of 3 independent experiments performed in duplicate, with t-test * P ≤ 0.05.
A.
PBS
EPS (24h)
B.
%G1: 67
%S: 20.1
%G2: 9.46
%Sub-G1: 5.47
Count
%G1: 76.7
%S: 8.53
%G2: 9.51
%Sub-G1: 5.37
Count
Count
Propidium Iodide →
Propidium Iodide →
B.
PBS
EPS (72h)
0.51
8.30
5.09
0.19
7-AAD
7-AAD
91.8
7.17
2.91
84.0
Annexin V (FITC) →
Annexin V (FITC) →

## Slide 5
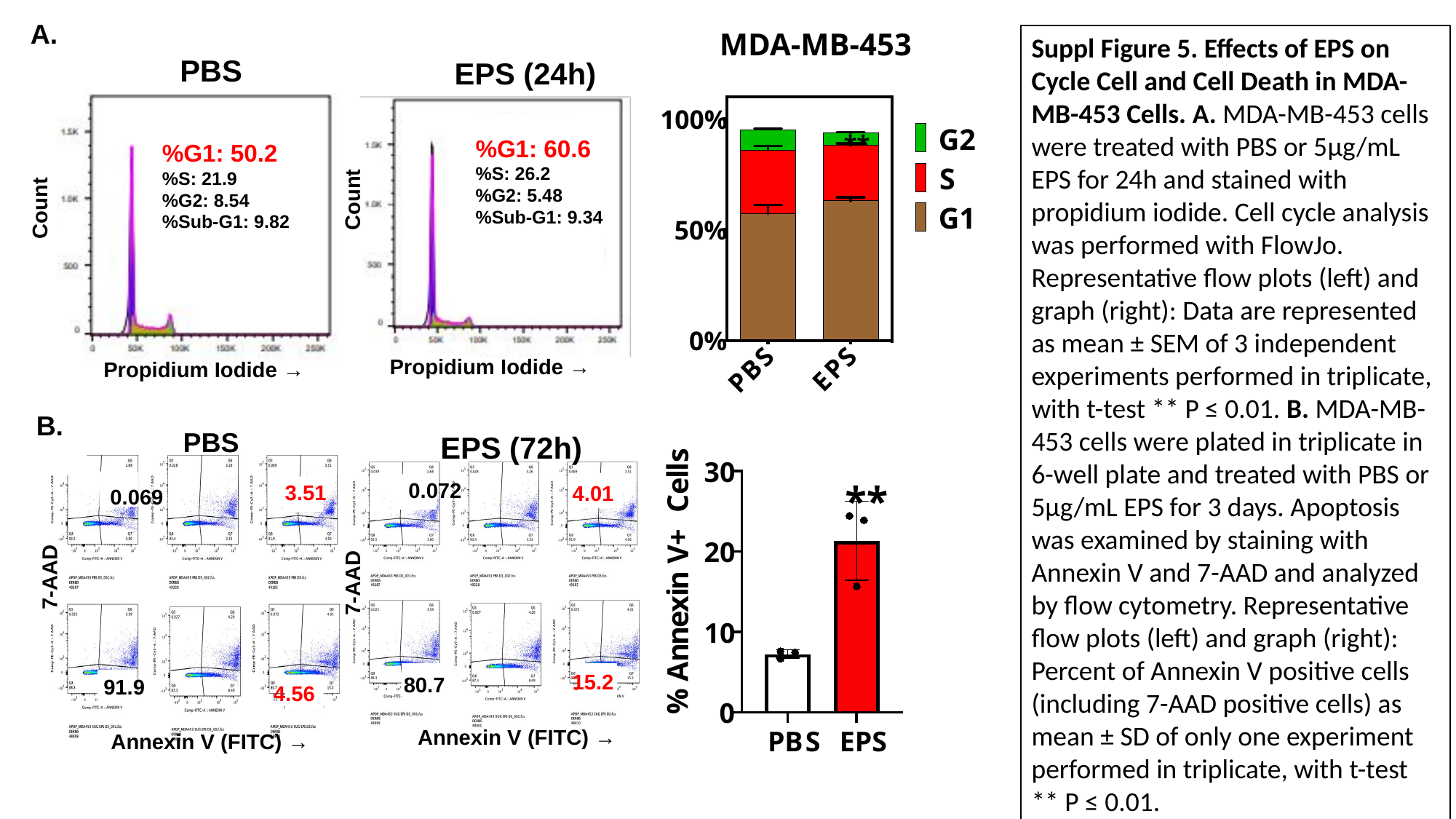

A.
MDA-MB-453
100%
G2
**
S
G1
50%
0%
S
S
P
B
E
P
PBS
EPS (24h)
%G1: 60.6
%S: 26.2
%G2: 5.48
%Sub-G1: 9.34
%G1: 50.2
%S: 21.9
%G2: 8.54
%Sub-G1: 9.82
Count
Count
Propidium Iodide →
Propidium Iodide →
B.
PBS
EPS (72h)
s
l
l
30
e
0.072
3.51
4.01
**
0.069
C
+
V
20
7-AAD
7-AAD
n
i
x
e
10
n
n
A
15.2
80.7
91.9
4.56
%
0
Annexin V (FITC) →
Annexin V (FITC) →
PB
S
EPS
Suppl Figure 5. Effects of EPS on Cycle Cell and Cell Death in MDA-MB-453 Cells. A. MDA-MB-453 cells were treated with PBS or 5μg/mL EPS for 24h and stained with propidium iodide. Cell cycle analysis was performed with FlowJo. Representative flow plots (left) and graph (right): Data are represented as mean ± SEM of 3 independent experiments performed in triplicate, with t-test ** P ≤ 0.01. B. MDA-MB-453 cells were plated in triplicate in 6-well plate and treated with PBS or 5μg/mL EPS for 3 days. Apoptosis was examined by staining with Annexin V and 7-AAD and analyzed by flow cytometry. Representative flow plots (left) and graph (right): Percent of Annexin V positive cells (including 7-AAD positive cells) as mean ± SD of only one experiment performed in triplicate, with t-test ** P ≤ 0.01.

## Slide 6
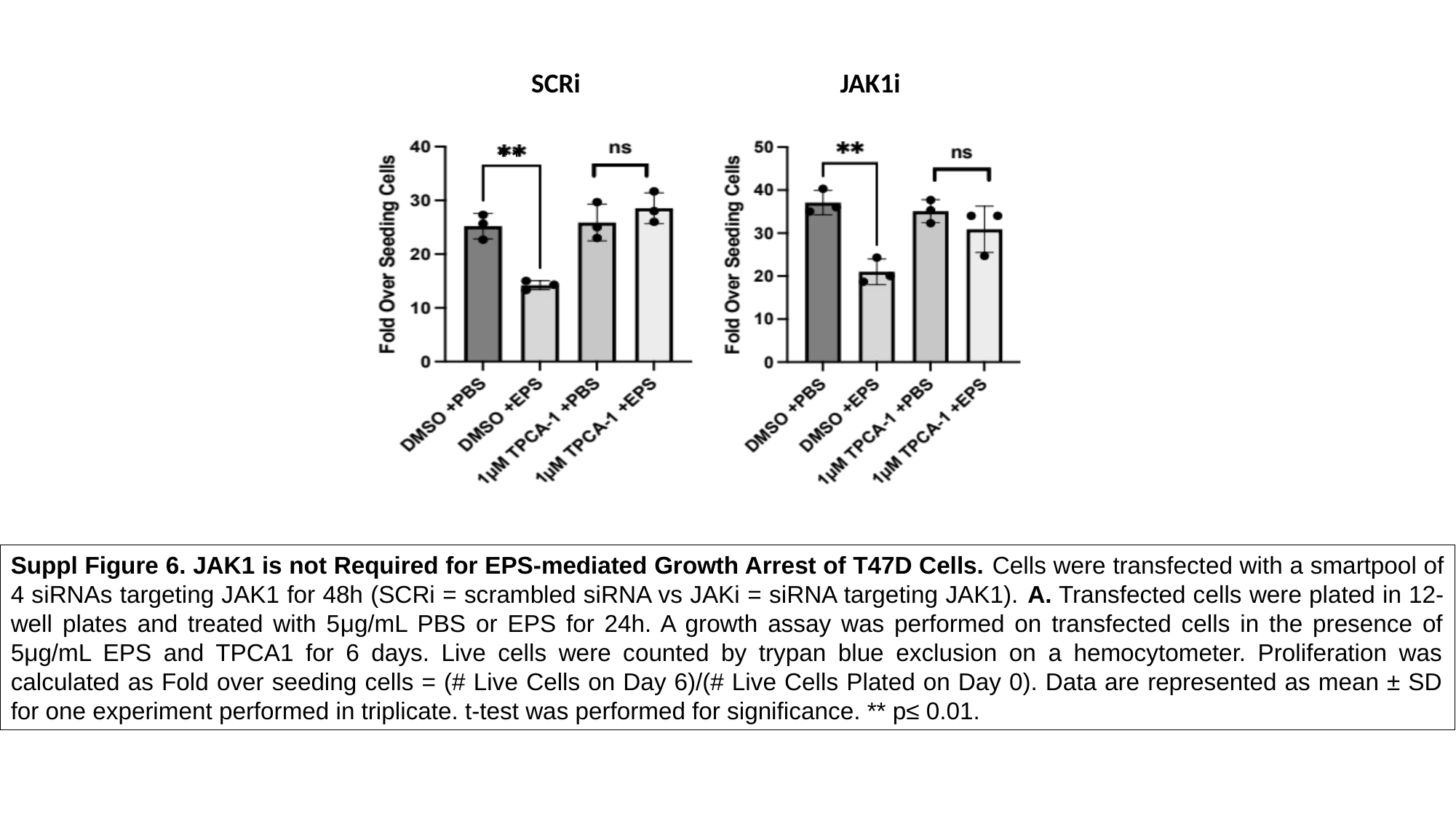

JAK1i
SCRi
**
Suppl Figure 6. JAK1 is not Required for EPS-mediated Growth Arrest of T47D Cells. Cells were transfected with a smartpool of 4 siRNAs targeting JAK1 for 48h (SCRi = scrambled siRNA vs JAKi = siRNA targeting JAK1). A. Transfected cells were plated in 12-well plates and treated with 5μg/mL PBS or EPS for 24h. A growth assay was performed on transfected cells in the presence of 5μg/mL EPS and TPCA1 for 6 days. Live cells were counted by trypan blue exclusion on a hemocytometer. Proliferation was calculated as Fold over seeding cells = (# Live Cells on Day 6)/(# Live Cells Plated on Day 0). Data are represented as mean ± SD for one experiment performed in triplicate. t-test was performed for significance. ** p≤ 0.01.

## Slide 7
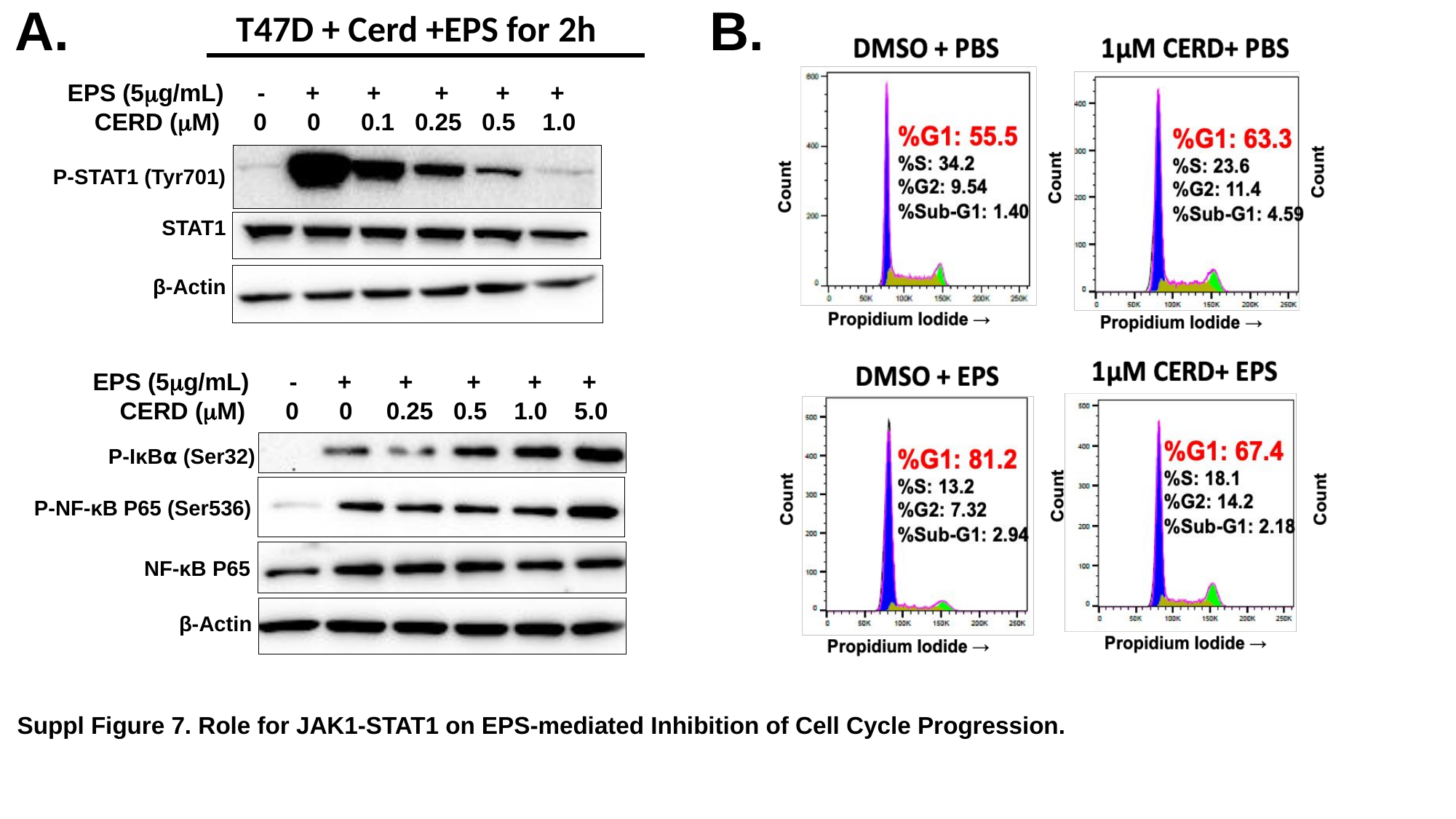

T47D + Cerd +EPS for 2h
A.
B.
EPS (5μg/mL) - + + + + +
 CERD (μM) 0 0 0.1 0.25 0.5 1.0
P-STAT1 (Tyr701)
STAT1
β-Actin
EPS (5μg/mL) - + + + + +
 CERD (μM) 0 0 0.25 0.5 1.0 5.0
P-IκB⍺ (Ser32)
P-NF-κB P65 (Ser536)
NF-κB P65
β-Actin
Suppl Figure 7. Role for JAK1-STAT1 on EPS-mediated Inhibition of Cell Cycle Progression.
